# Supplementary material for: A versatile functionalized ionic liquid to boost the solution-mediated performances of lithium-oxygen batteries
Source: Nat Commun. 2019 Feb 5;10:602. doi: 10.1038/s41467-019-08422-8 (PMC6363722; doi:10.1038/s41467-019-08422-8)
Supplement: Supplementary file 1 — Supplementary Information [file 41467_2019_8422_MOESM1_ESM.pdf]

Supplementary Information

A versatile functionalized ionic liquid to comprehensively boost the solution-mediated performances of Li-oxygen batteries

By Zhang *et al.*

Supplementary Figures

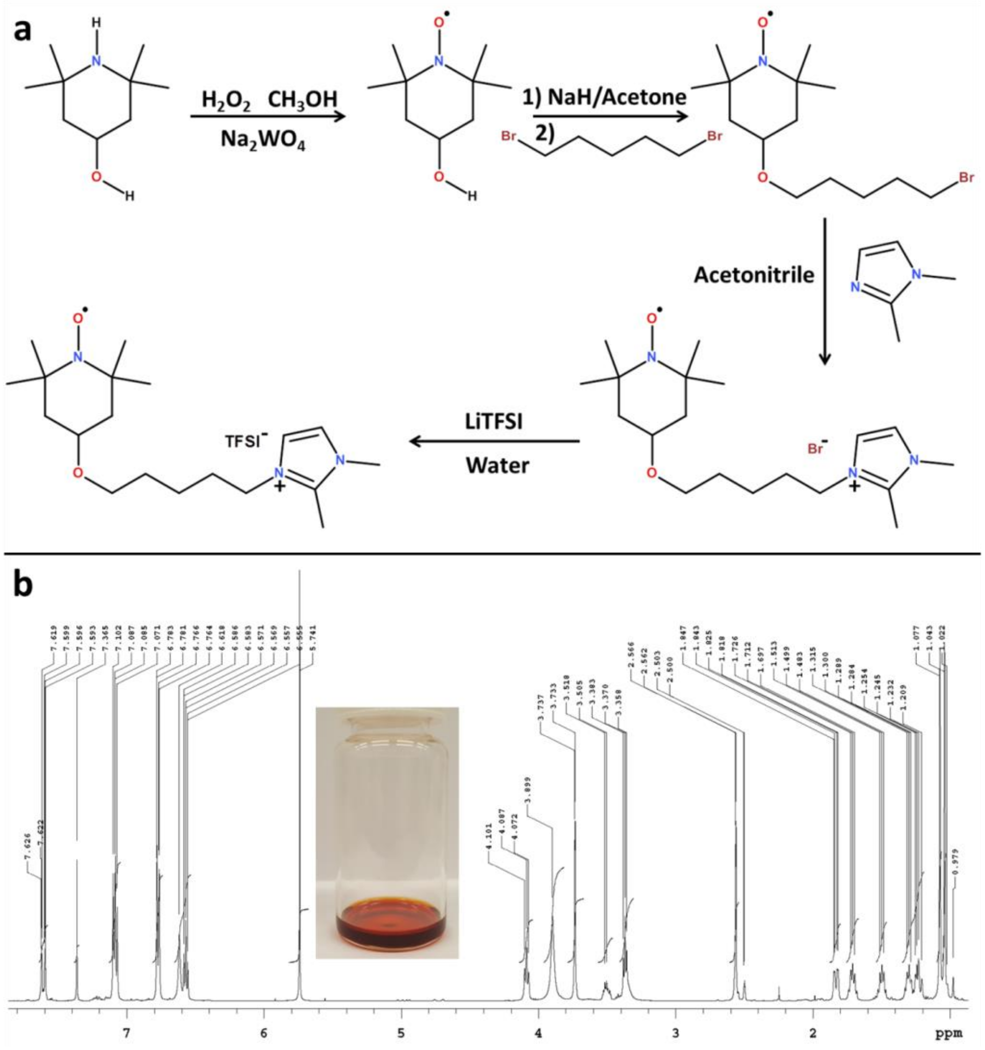

Supplementary Figure 1 | Synthesis route and ^{1}H NMR spectrum of the IL-TEMPO material. ^{1}H NMR (DMSO-d_6, ppm): *d* = 1.04 (s, 6H, $(C\underline{H}_3)_{Pyp}$), 1.08 (s, 6H, $(C\underline{H}_3)_{Pyp}$), 1.19–1.34 (m, 4H), 1.46–1.52 (m, 2H), 1.67–1.75 (m, 2H), 1.80–1.87 (m, 2H), 2.54–2.59 (m, 3H, $C(2)_{Im}$-$C\underline{H}_3$), 3.37 (t, 2H, ^{3}J(H,H) = 6.4 Hz, -O-$C\underline{H}_2$-), 3.45–3.55 (m, 1H, $C(4)_{Pyp}$-$\underline{H}$), 3.71–3.76 (m, 3H, $N(1)_{Im}$-$C\underline{H}_3$), 4.09 (t, 2H, ^{3}J(H,H) = 7.6 Hz, -$C\underline{H}_2$-N≤), 7.58–7.61 (m, 1H, $C(4)_{Im}$-$\underline{H}$ or $C(5)_{Im}$-$\underline{H}$), 7.61–7.64 (m, 1H, $C(4)_{Im}$-$\underline{H}$ or $C(5)_{Im}$-$\underline{H}$)[1,2]. The inset image is the digital photo of the as-prepared IL-TEMPO.

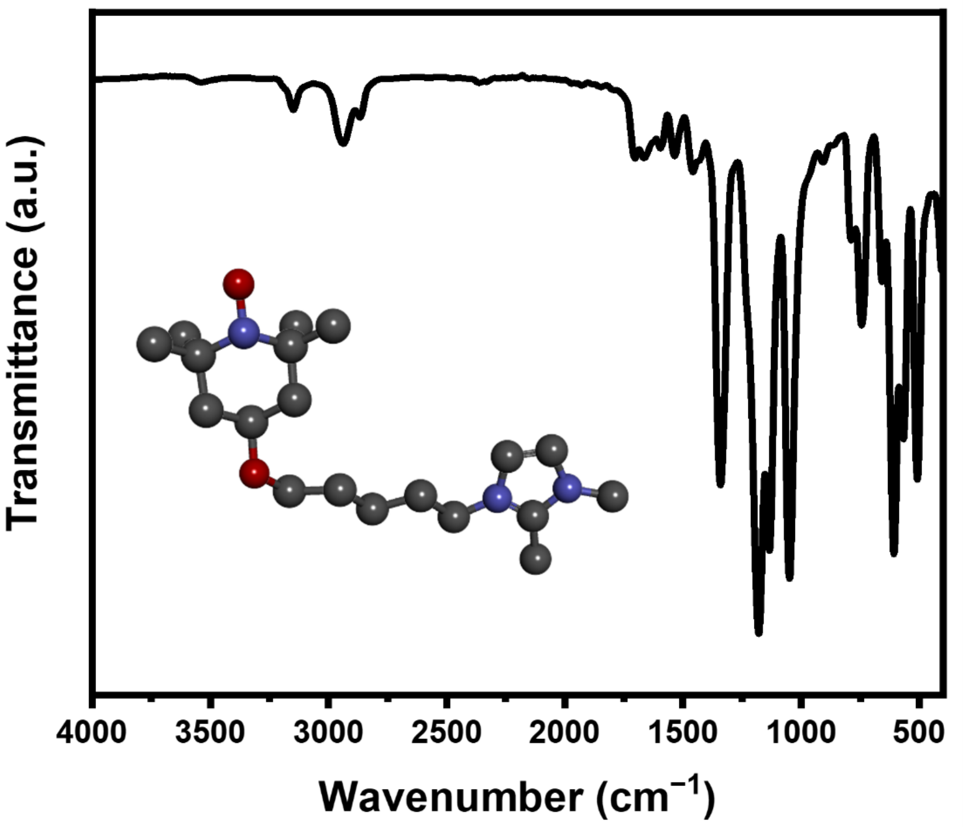

Supplementary Figure 2 | FTIR spectrum of the IL-TEMPO. The FTIR of IL-TEMPO shows the characteristic peaks of TFSI anion and imidazolium ionic liquids. FTIR ATR (*n*, cm^{-1}) is analysed as: 571 (CF_3 asym bend), 610, 615 (SO_2 asym bend), 650 (S-N-S bend), 741 (CF_3 sym bend), 762, 789 (skeletal asym bend of imidazolium ring), 1056 (S-N-S asym str, skeletal asym str of imidazolium ring, C-C asym str, N-CH_3 twisting, aliphatic ether C-O str), 1136 (SO_2 sym str), 1180 (N-CH_2 and N-CH_3 C–N str, C–C str, CF_3 asym str), 1226 (CF_3 sym str), 1331, 1348 (SO_2 asym str), 1462, 1539, 1590 (skeletal str of imidazolium ring), 2869, 2939, 2976 (sp^3 C-H str), 3148 (sp^2 C-H str).

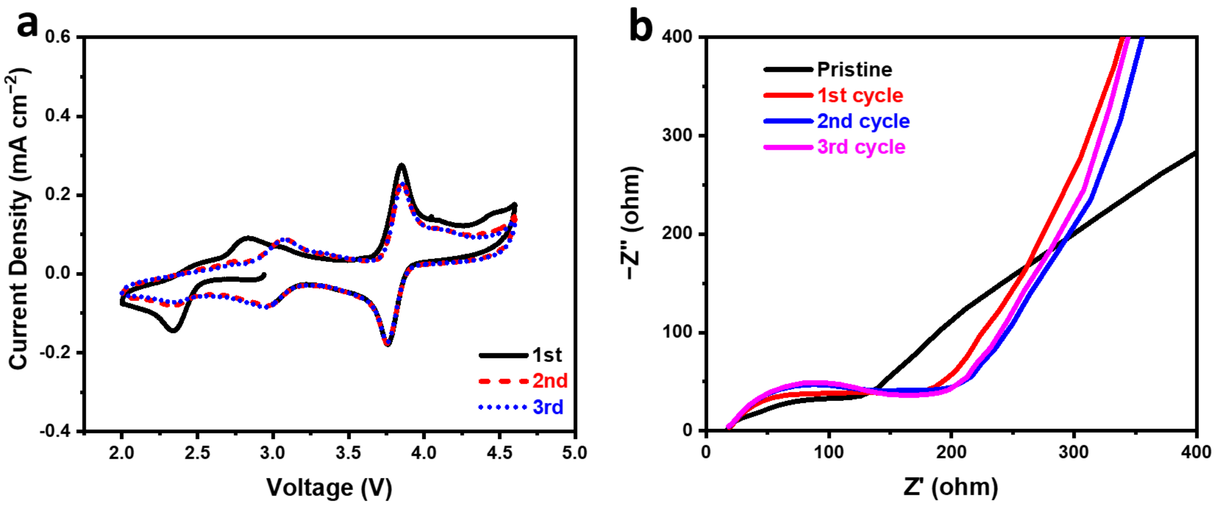

Supplementary Figure 3 | The CV curves and impedance spectra of the cell with IL-TEMPO electrolyte in argon atmosphere at different cycles. The lithium foil was directly used as the anode without any pre-treatment. The scan rate is 0.5 mV s^{-1}.

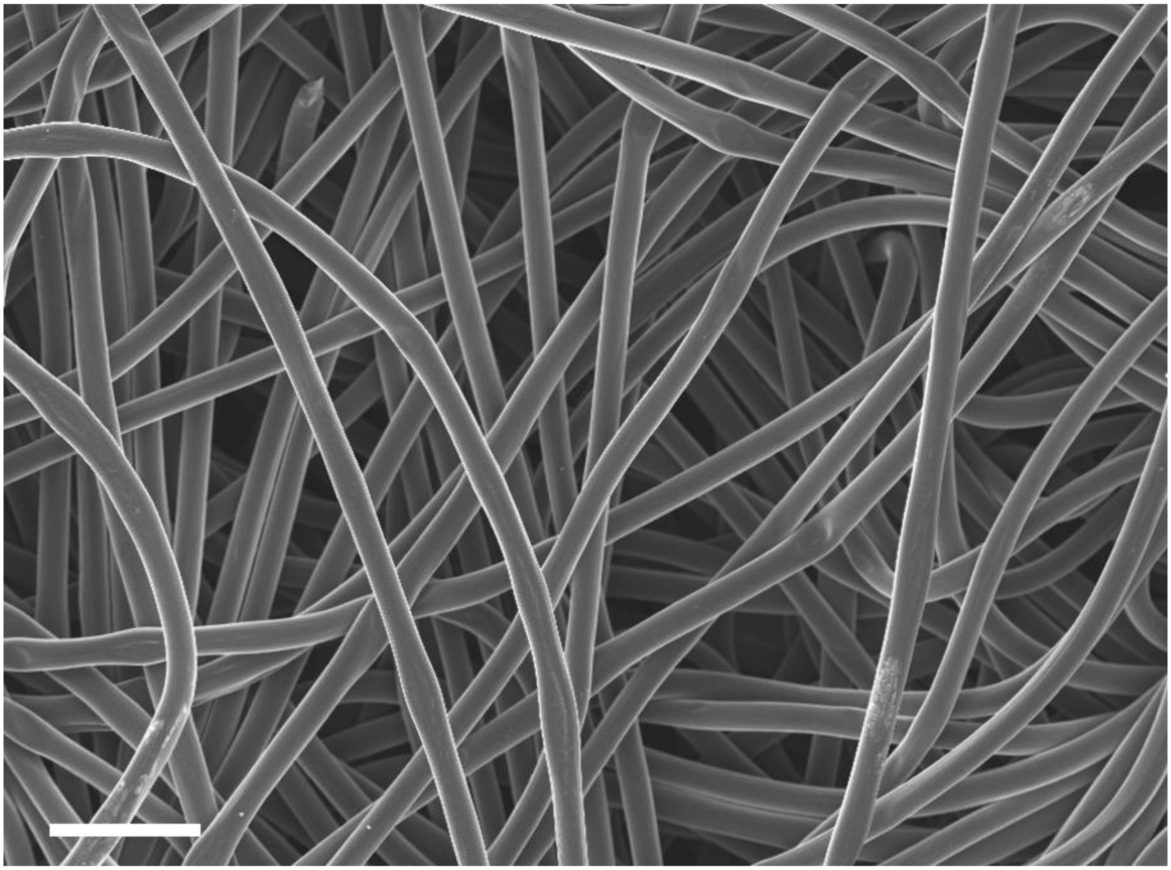

Supplementary Figure 4 | SEM image of the carbon paper. Scale bar is 50 µm.

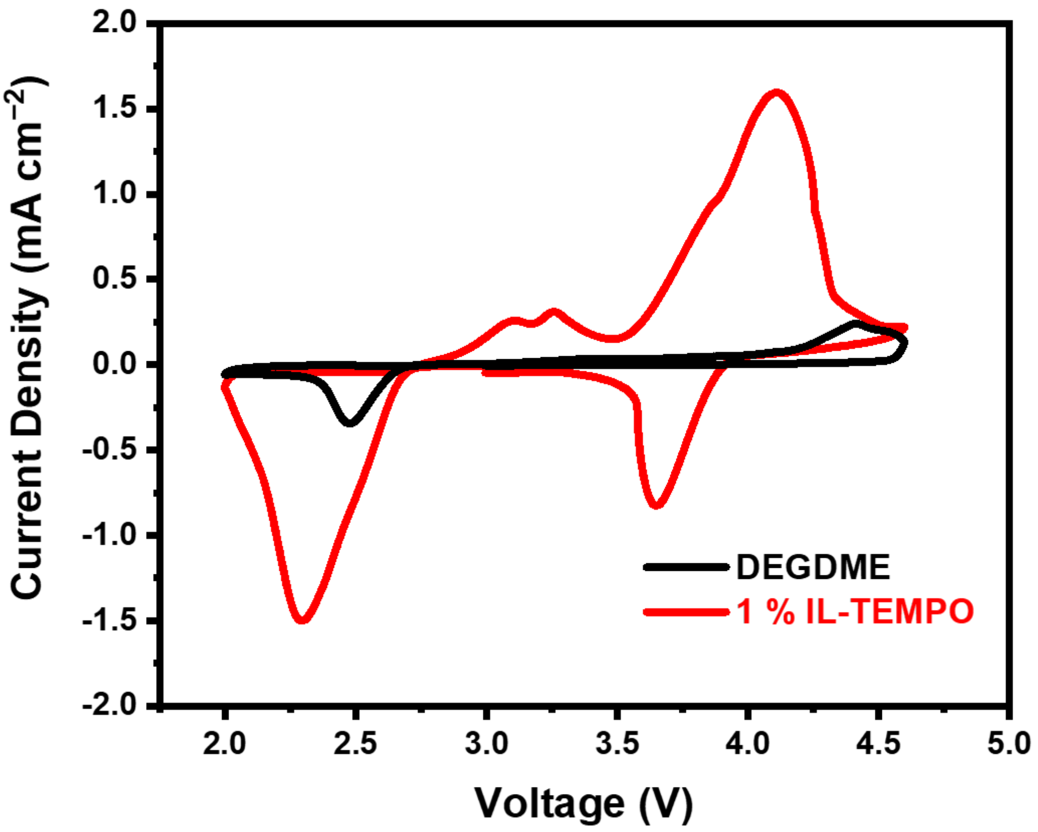

Supplementary Figure 5 | Cyclic voltammetry test of the IL-TEMPO electrolyte in oxygen atmosphere. The scan rate is 0.5 mV s^{-1}. The redox peaks lie in the voltage where p-/n-doping of TEMPO functional group ocurrs[1]. The peaks of Li-O_2 cell with IL-TEMPO electrolyte are consistent with the one in argon atmosphere (Figure 1b).

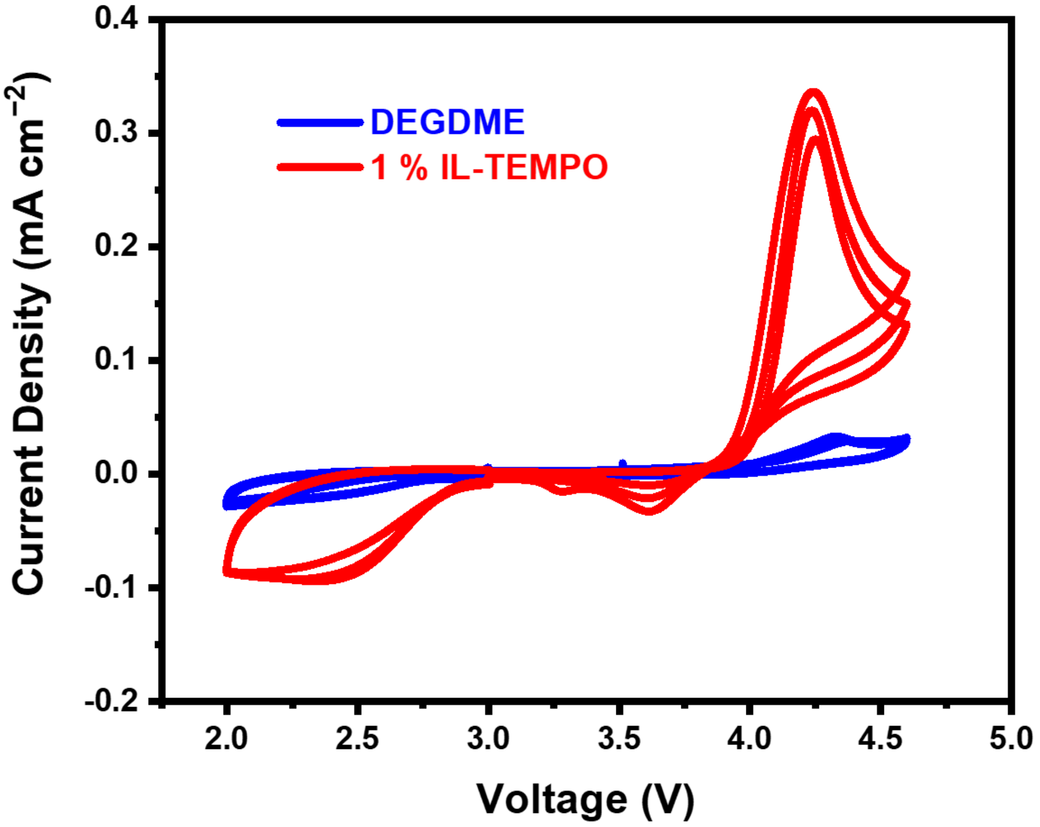

Supplementary Figure 6 | Cyclic voltammetry test of the IL-TEMPO electrolyte in oxygen atmosphere with only current collector as the air cathod. The scan rate is 0.5 mV s^{-1}.

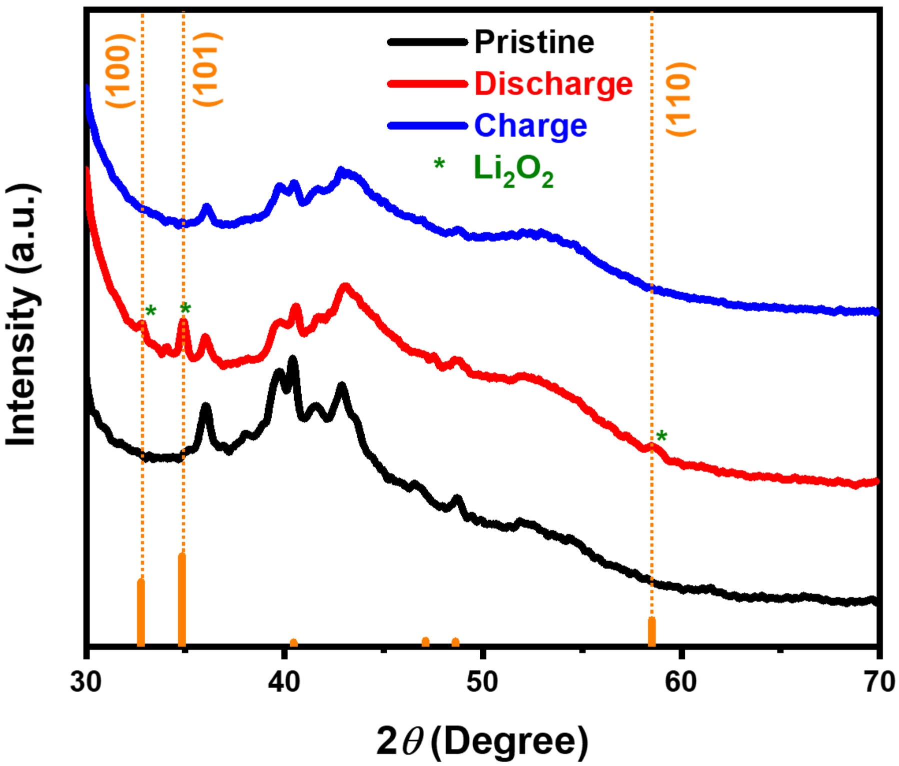

Supplementary Figure 7 | Post-mortem characterization of the discharged electrodes when the IL-TEMPO electrolyte was used. XRD spectra of the electrodes before discharge, after discharge, and after charge. The patterns are indexed with the standard Li_2O_2 peaks. The result clearly indicates that Li_2O_2 is the main discharge product.

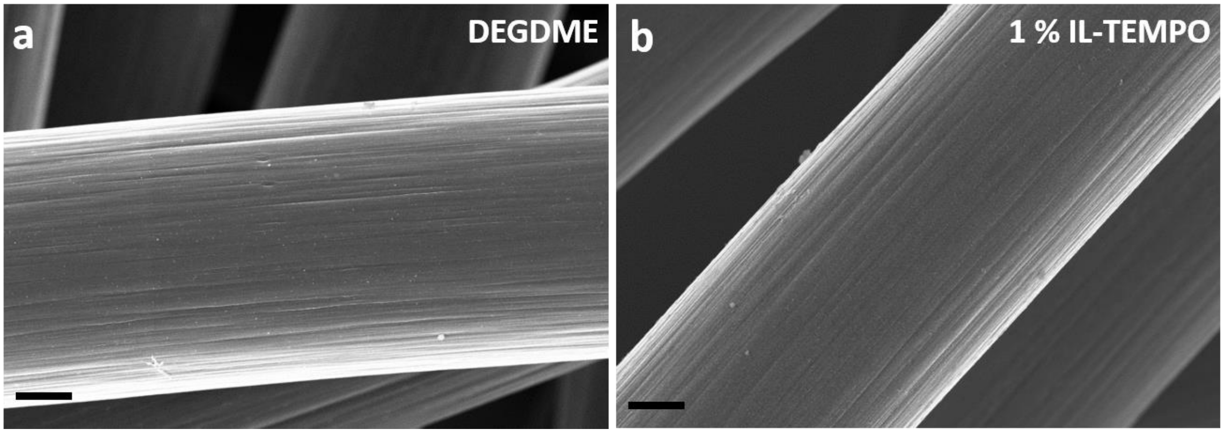

Supplementary Figure 8 | The SEM images of the charged carbon paper electrode from Li-O_2 batteries with a. DEGDME electrolyte and b. 1 % IL-TEMPO electrolyte. Scale bars are 2 μm.

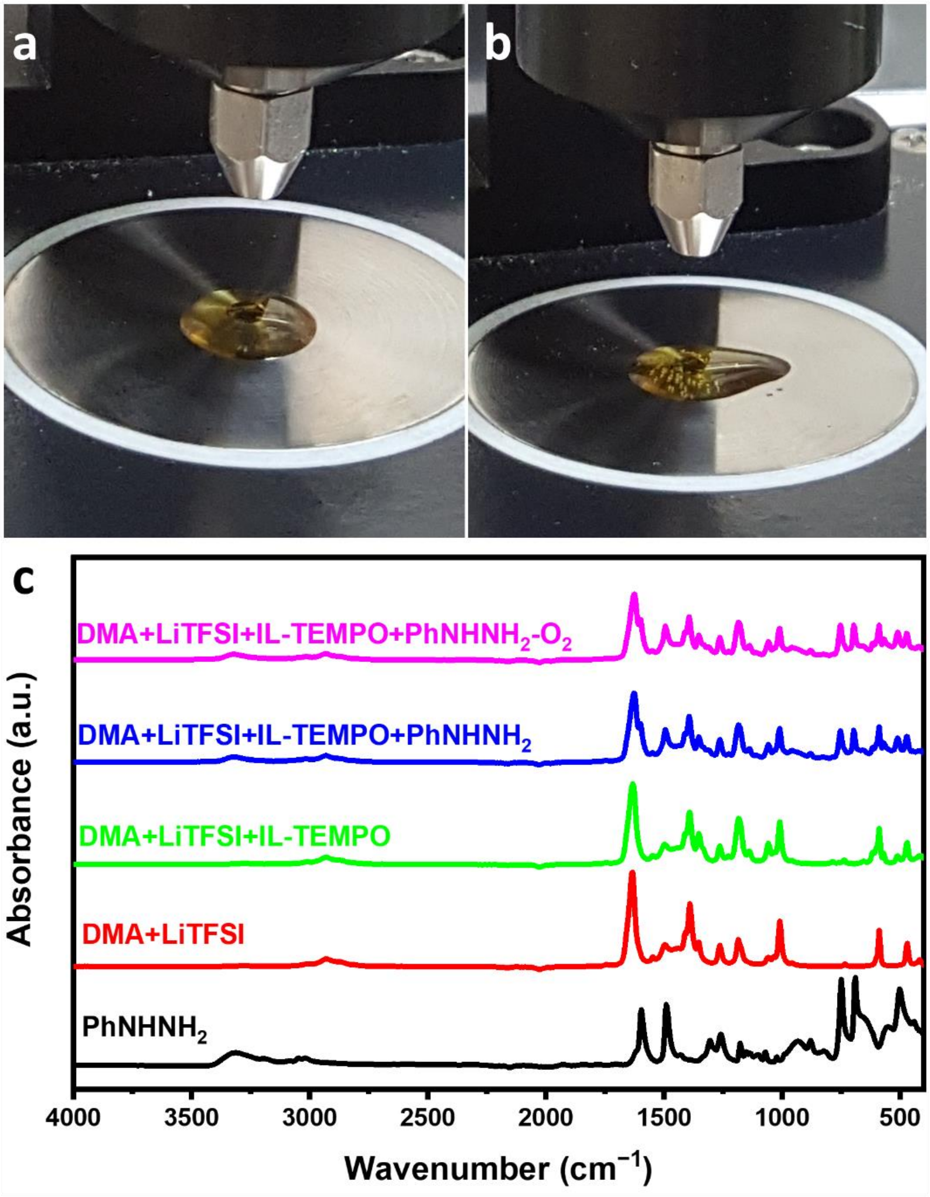

Supplementary Figure 9 | The mechanism experiments demonstrating the interactions between reduced IL-TEMPO and oxygen. a. The digital photo of the argon group (DMA+LiTFSI+IL-TEMPO+$PhNHNH_2$) when casted to the FTIR crystal. **b.** The digital photo of the oxygen group (DMA+LiTFSI+IL-TEMPO+$PhNHNH_2$-O_2) when casted to the FTIR crystal. **c.** The corresponding FTIR spectra of the mixtures.

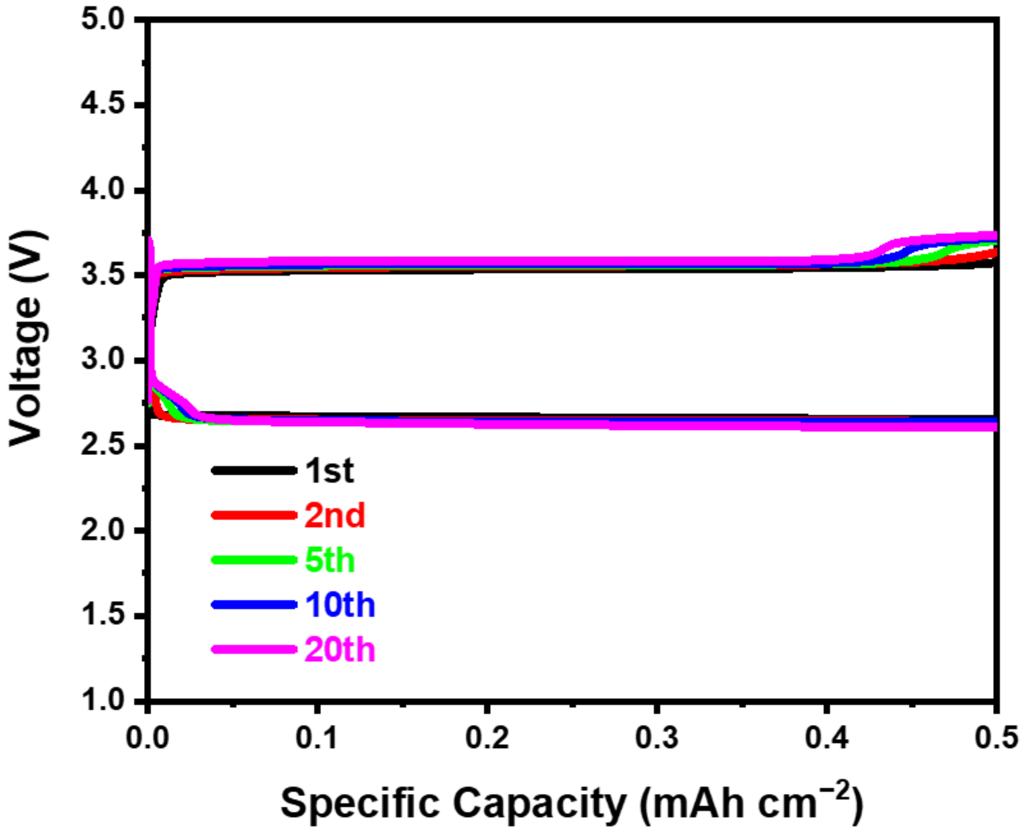

Supplementary Figure 10 | The electrochemical performance of the Li-O_2 battery with carbon paper electrode. The discharge-charge profiles of the Li-O_2 battery with carbon paper electrode. The current density was 0.1 mA cm^{-2}.

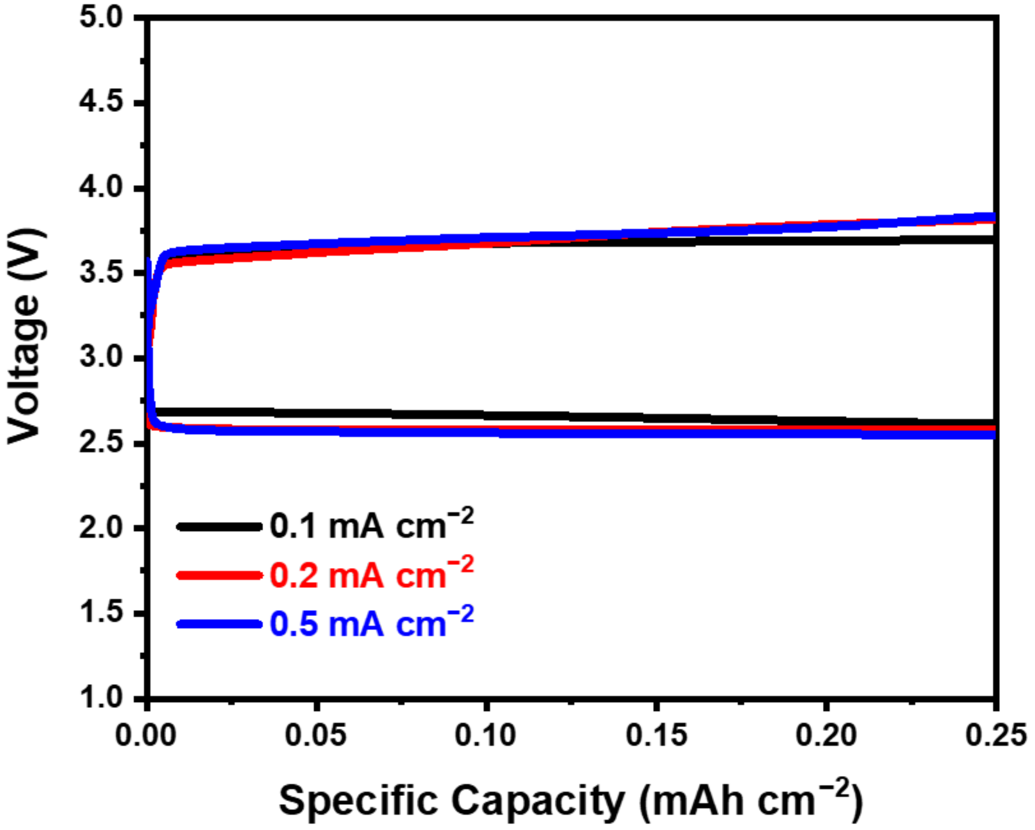

Supplementary Figure 11 | The rate performances of the Li-O_2 battery with carbon paper electrode.

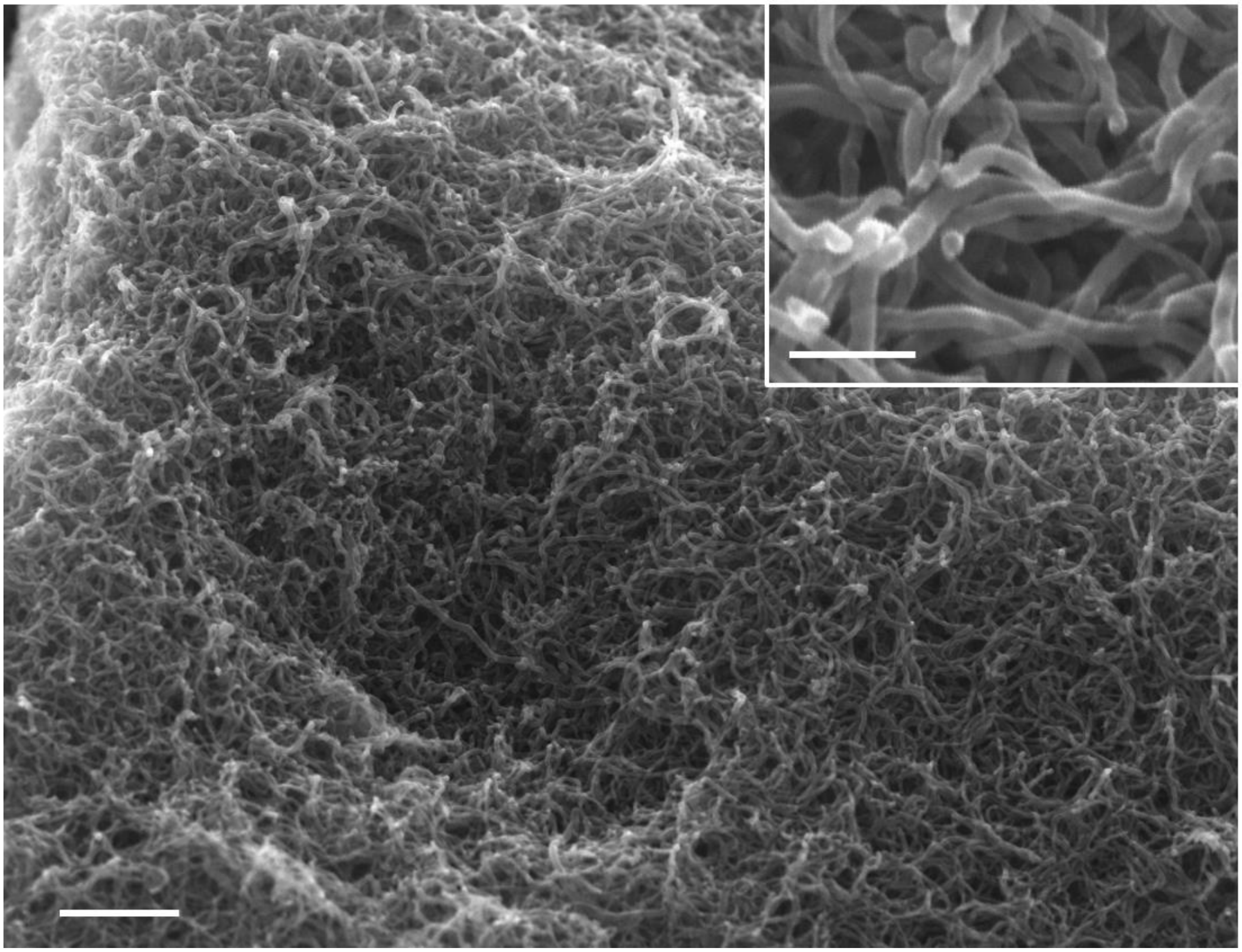

Supplementary Figure 12 | SEM images of the CNT film cathode. Scale bar is 1 µm. The inset image is the CNT film at higher magnification. Scale bar is 200 nm.

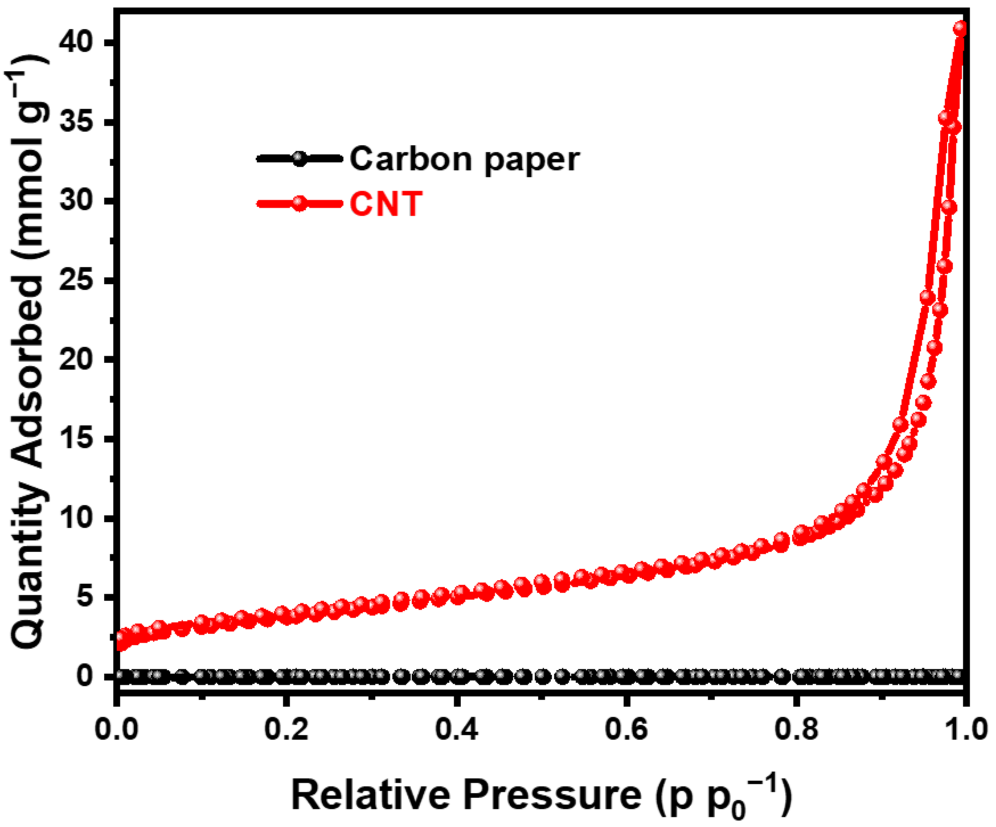

Supplementary Figure 13 | BET results of the carbon paper and CNT. The BET area of carbon paper is obtained as 0.984 m^2 g^{-1}. The BET surface area of CNT is as high as 315.12 m^2 g^{-1}. Moreover, the pore volume of CNT (0.4526 cm^3 g^{-1}) is more than 100 times of carbon paper (0.0042 cm^3 g^{-1}). Therefore, the replacement of carbon paper with CNT should generate higher discharge capacity when regular DEGDME electrolyte is used, which should provide more insight by comparing it with the IL-TEMPO electrolyte.

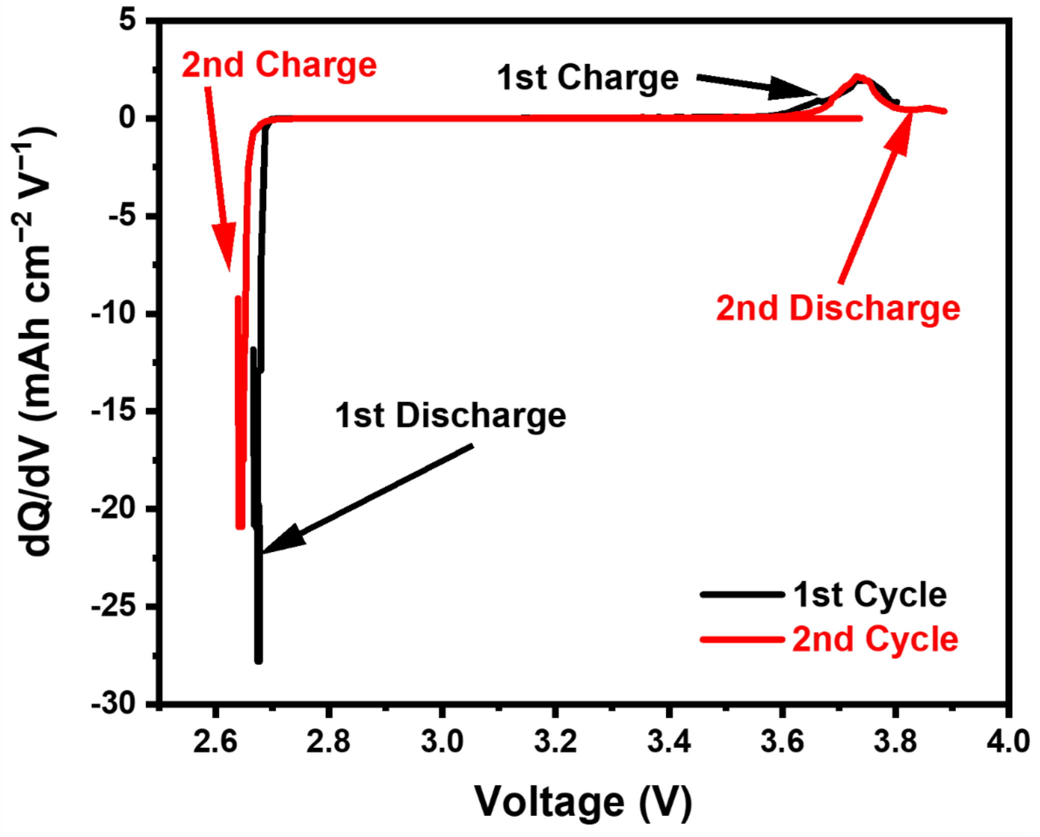

Supplementary Figure 14 | The calculated dQ/dV *vs.* V profiles of the IL-TEMPO containing Li-O_2 battery. The data was obtained by calculating the discharge-charge result from Figure 3b.

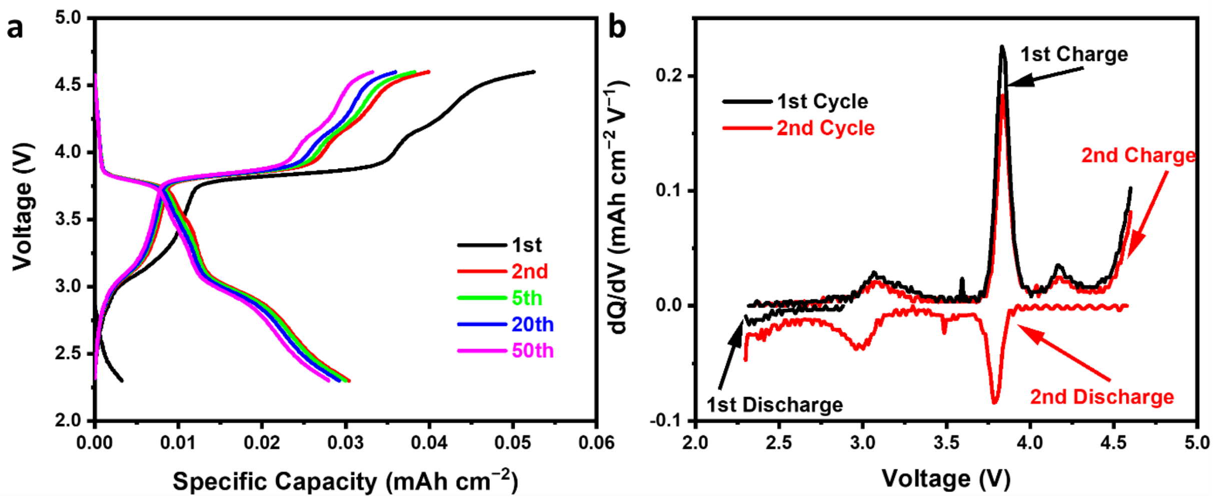

Supplementary Figure 15 | The electrochemical profiles of the two-electrode cell with IL-TEMPO containing electrolyte in argon atmosphere. a. The discharge-charge curves of the cell. The current density was 0.1 mA cm^{-2}. **b.** The calculated dQ/dV vs. V profiles based on **a.** The calculated result is consistent with the CV curves in Figure 1b.

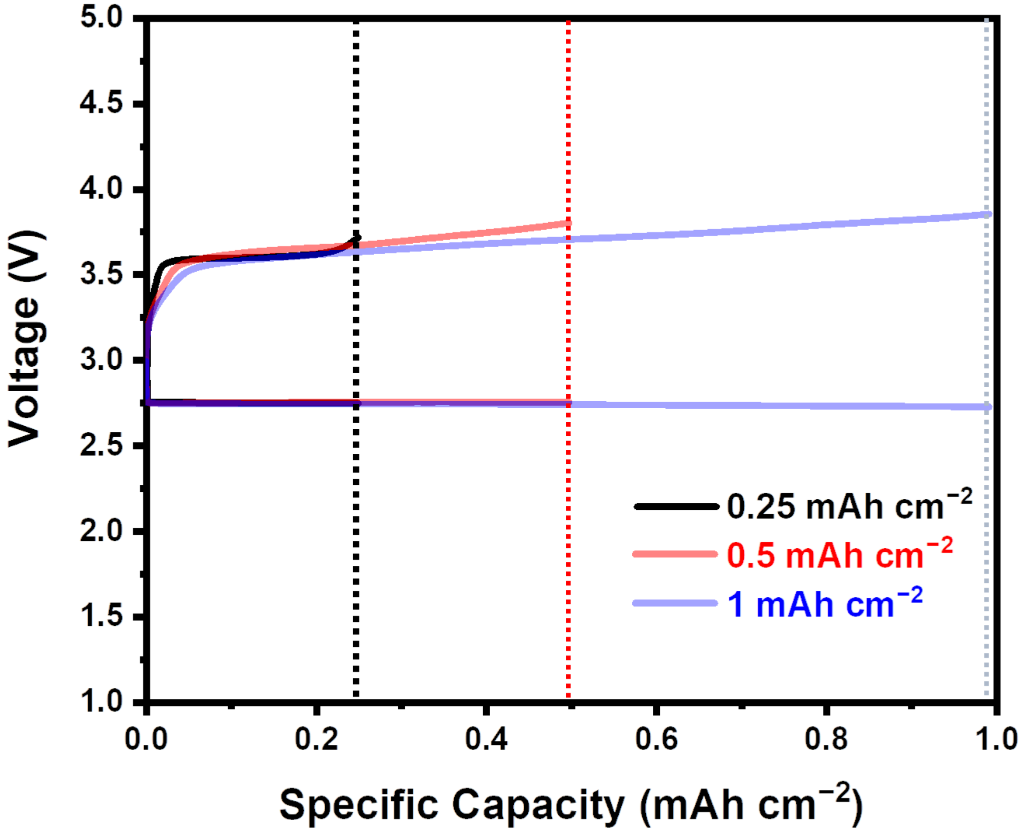

Supplementary Figure 16 | The discharge-charge curves of Li-O_2 batteries at different discharge depths. The results indicate the charge processes mainly involved the decomposition of Li_2O_2 other than the self-shuttle of IL-TEMPO mediator.

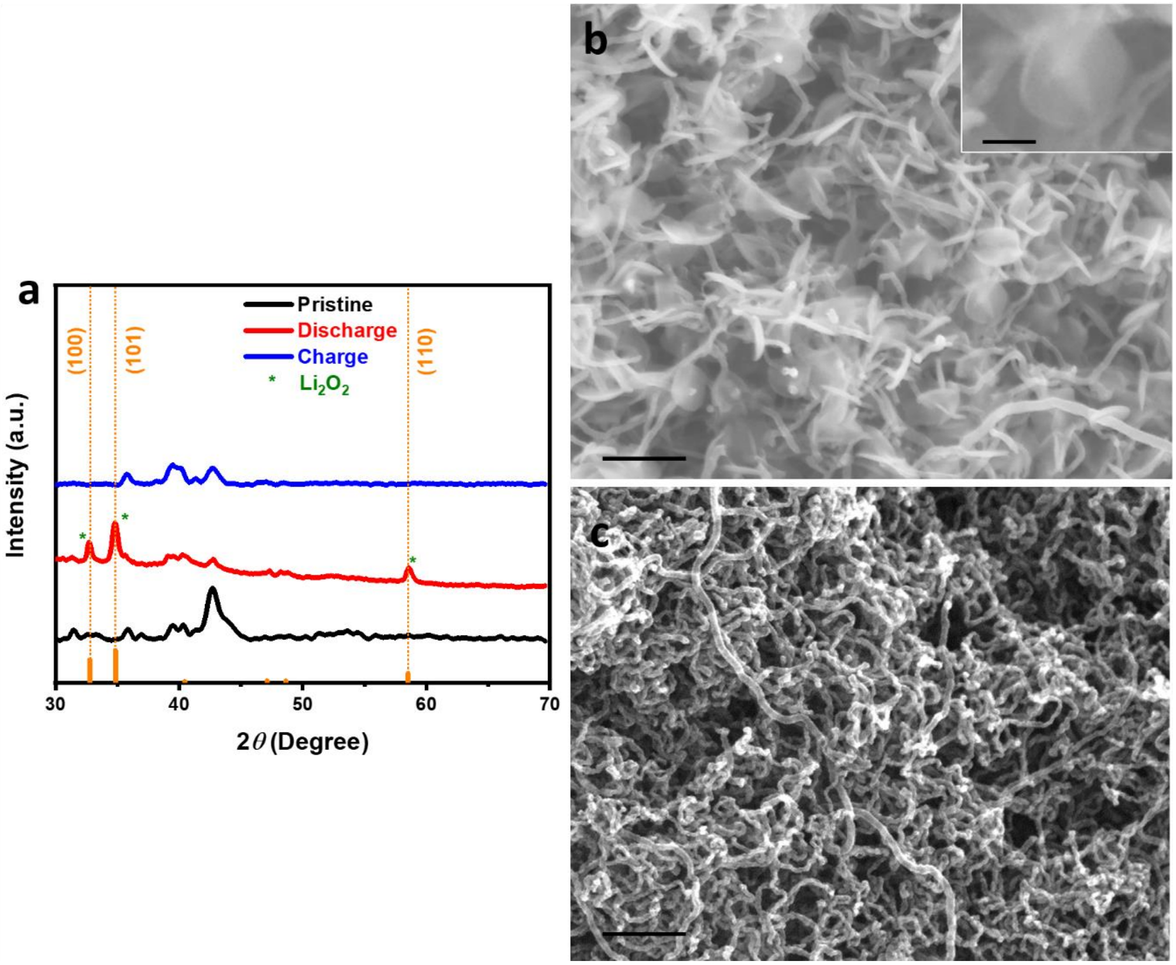

Supplementary Figure 17 | The characterization data of the electrodes when the IL-TEMPO electrolyte was used. a. XRD spectra of the electrodes before and after first cycle. The spectra are indexed with the standard Li_2O_2 peaks. **b.** The SEM image of the discharged electrode. Scale bar is 400 nm. The inset image is the one with high magnification. Scale bar is 100 nm. **c.** The SEM image of the charged electrode. Scale bar is 400 nm.

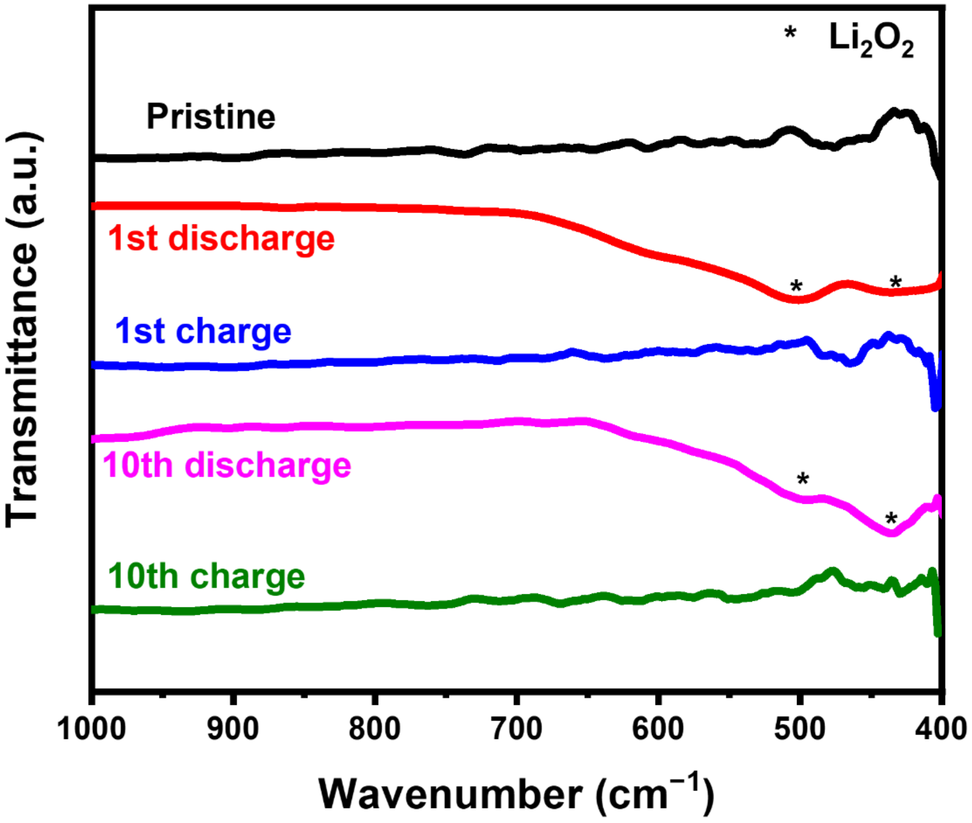

Supplementary Figure 18 | FTIR spectra of the electrode before and after cycles using 1 % IL-TEMPO electrolyte. The cells were operated in O_2 atmosphere.

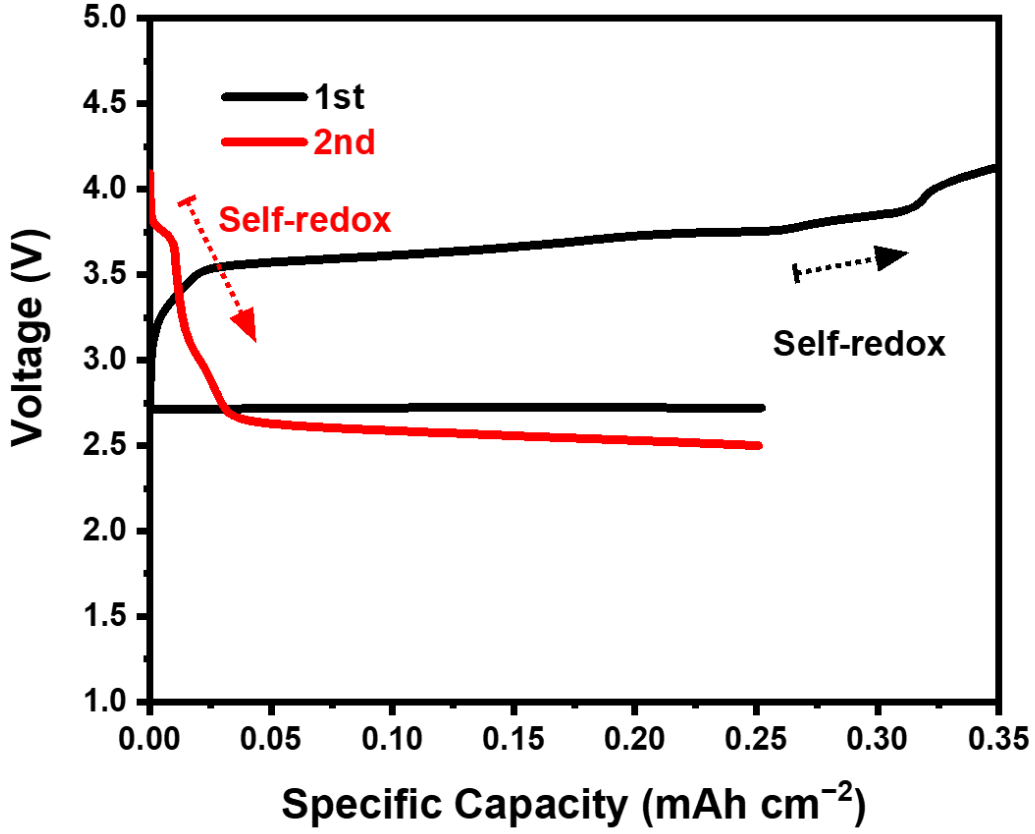

Supplementary Figure 19 | The discharge-overcharge profiles of Li-O_2 cell with 1 % IL-TEMPO electrolyte. The current density was 0.1 mA cm^{-2}.

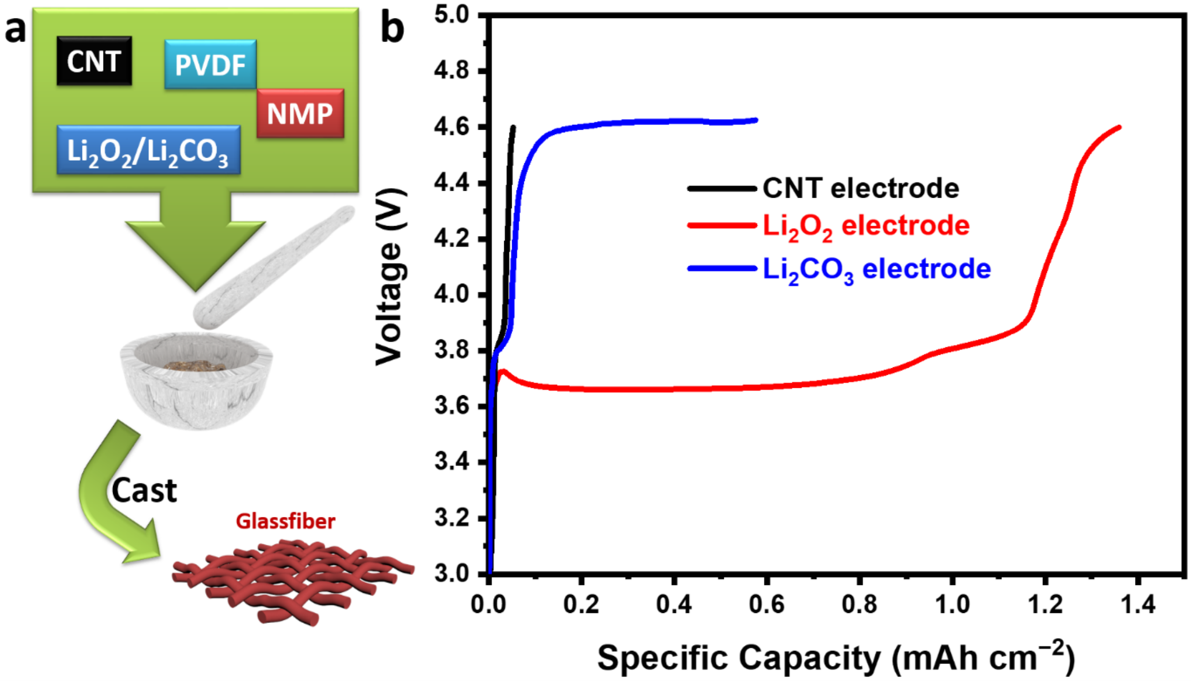

Supplementary Figure 20 | The demonstration experiment for proving the catalytic capability of IL-TEMPO redox mediator towards different discharge products. a. The schematic illustration of the preparation of discharge products-containing cathode. **b.** The corresponding charge curves of the cathodes using different additives. The current density was 0.1 mA cm^{-2}. The results indicate that the IL-TEMPO could catalyze the decomposition of Li_2O_2 other than crystalline Li_2CO_3.

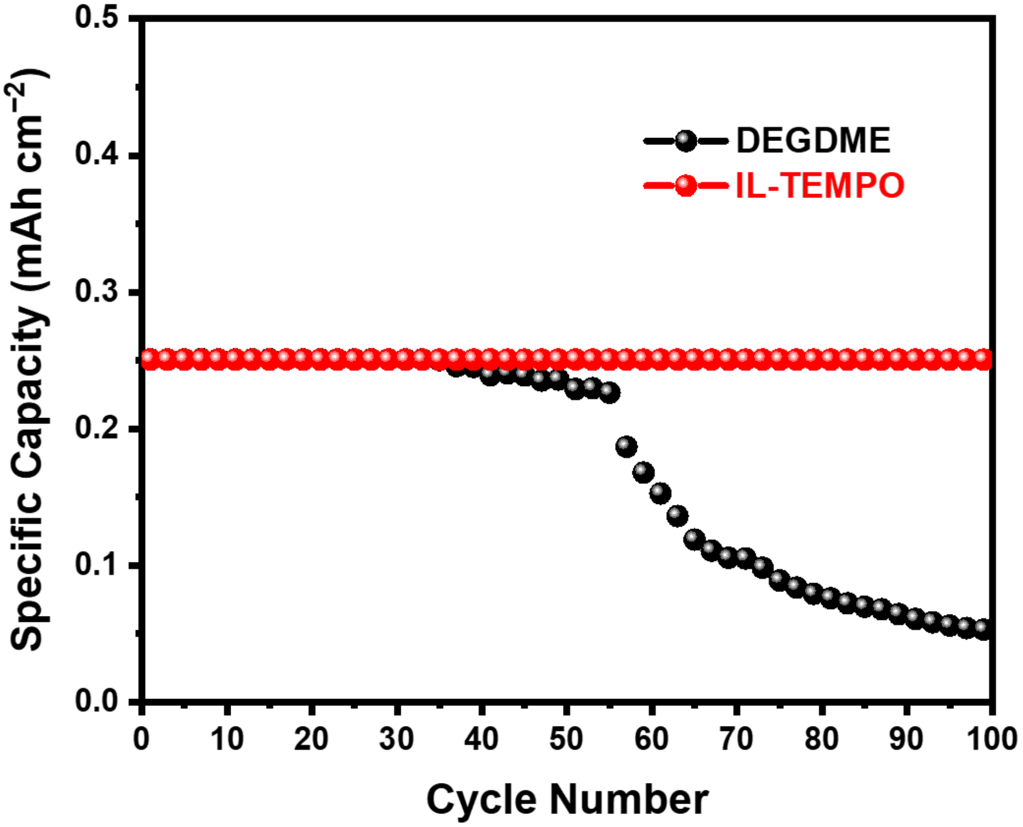

Supplementary Figure 21 | Cycling performance of the Li-O_2 cell with CNT electrode. The electrolyte used contains 1 % of IL-TEMPO. The current density was 0.1 mA cm^{-2}.

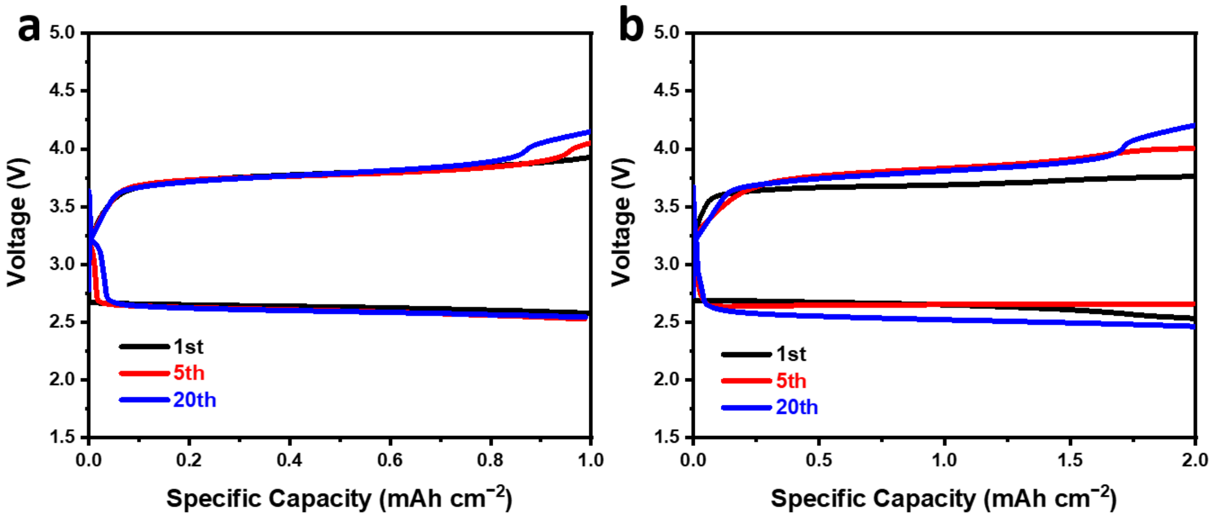

Supplementary Figure 22 | The discharge-charge profiles of Li-O_2 batteries with 1 % IL-TEMPO electrolyte with extended capacity restriction. a. The capacity was restricted to 1 mAh cm^{-2}. The current density was 0.1 mA cm^{-2}. **b.** The capacity was restricted to 2 mAh cm^{-2}. The current density was 0.2 mA cm^{-2}.

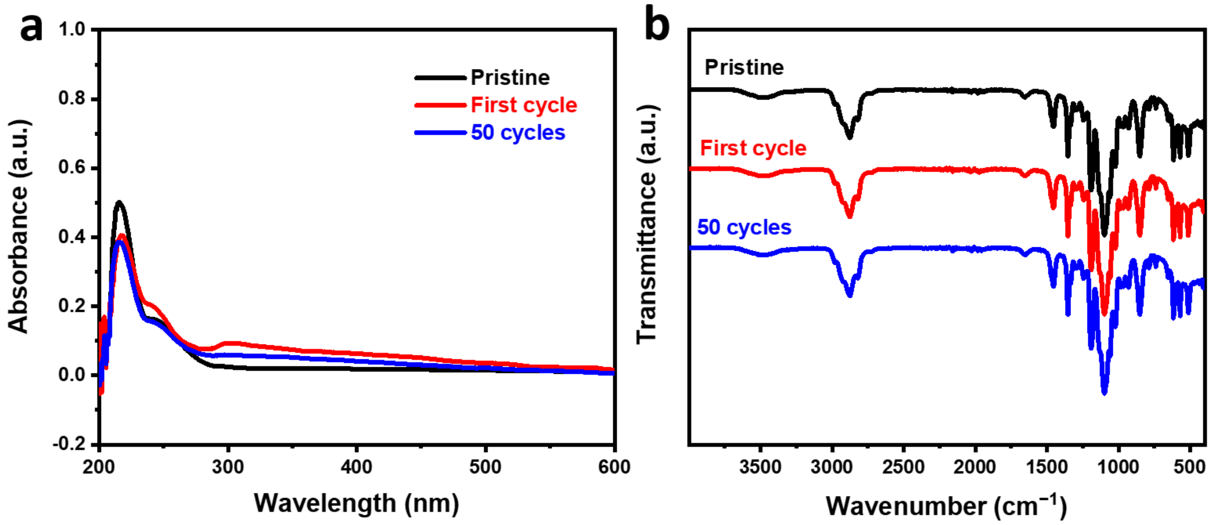

Supplementary Figure 23 | Characterization of IL-TEMPO after cycling. a. UV spectra comparison of the pristine electrolyte and the electrolytes after the first cycle (discharge-charge) and 50 cycles. **b.** The corresponding FTIR spectra of the electrolytes.

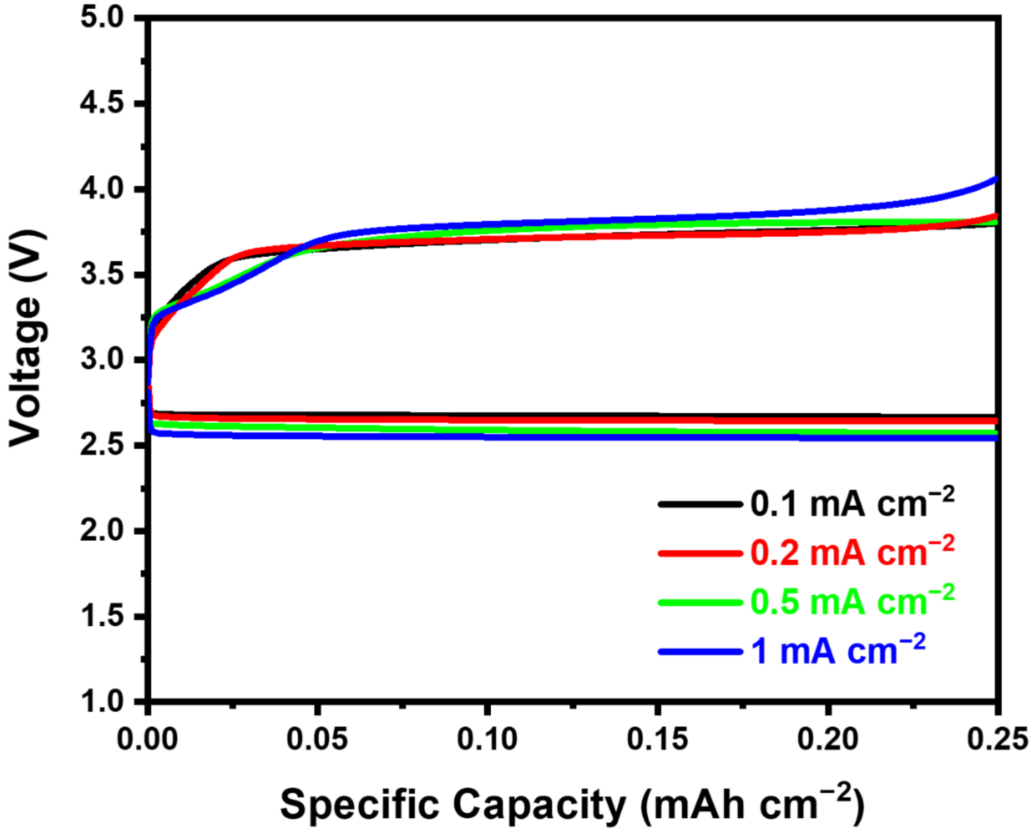

Supplementary Figure 24 | The rate performance of Li-O_2 batteries with IL-TEMPO (1 %) electrolyte. The electrolyte used was 1 % IL-TEMPO electrolyte.

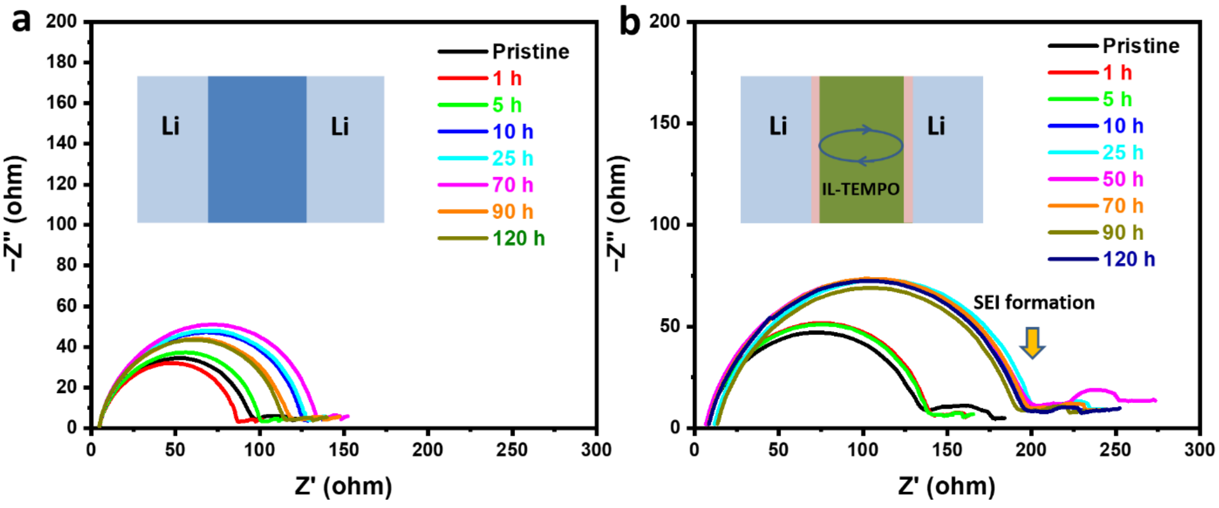

Supplementary Figure 25 | The lithium stability investigation in Li|Li symmetric cells in argon atmosphere. a. Impedance spectra of the symmetric cell with DEGDME electrolyte. **b.** Impedance spectra of the symmetric cell with IL-TEMPO (1 %) containing electrolyte. The cells were assembled and rest for 120 h. The impedance tests were taken at certain time period.

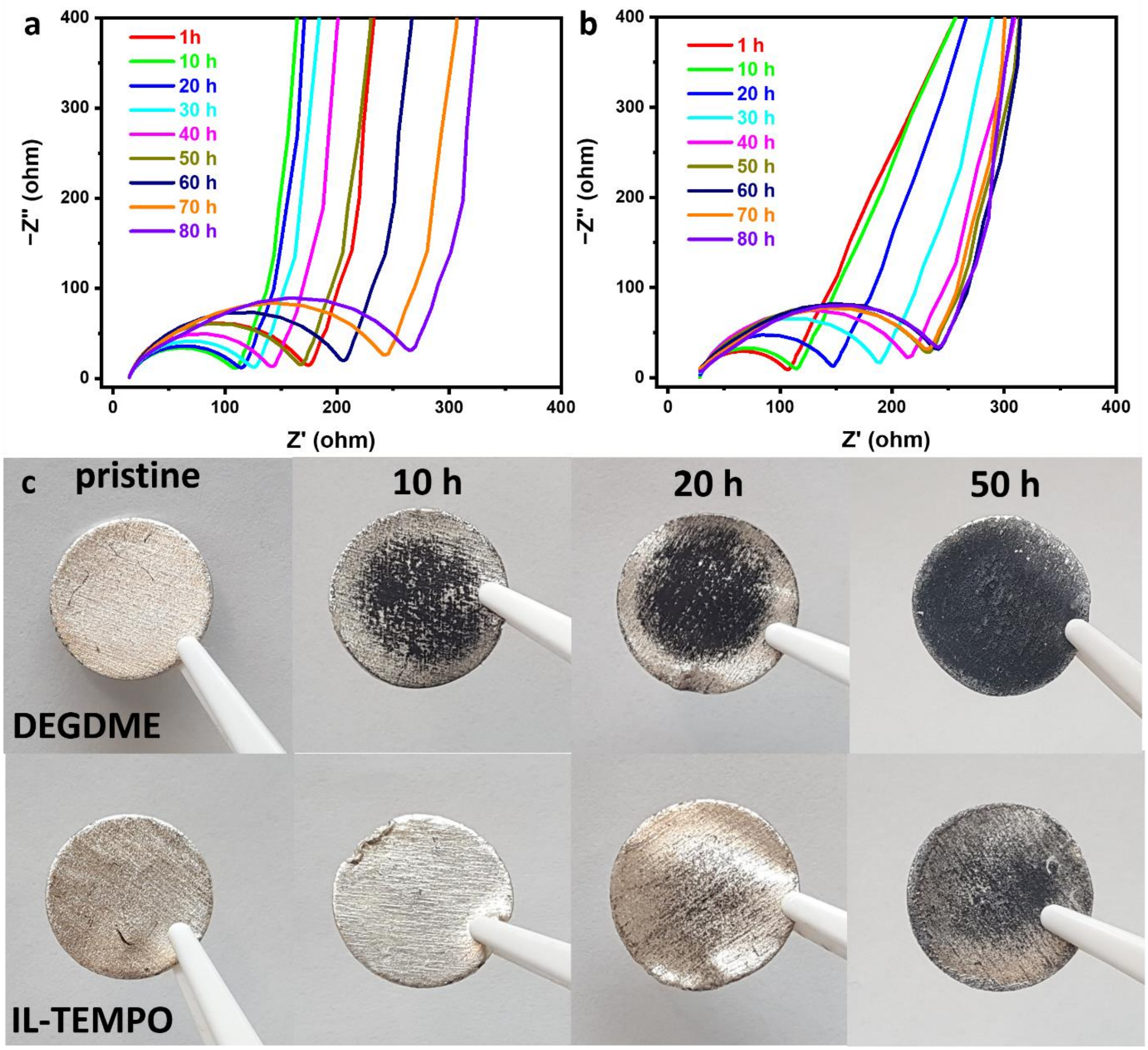

Supplementary Figure 26 | The impedance spectra of the two-electrode Li-O_2 cells when rested at different time period in oxygen atmosphere and corresponding photos of lithium anodes. Supplementary Figure 26a was obtained using DEGDME electrolyte, while Supplementary Figure 26b was using IL-TEMPO (1 %) contained electrolyte. The results show that the impedance in DEGDME electrolyte was continuously increasing due to the continuous corrosion of lithium anode in oxygen atmosphere, while the one in IL-TEMPO contained electrolyte became steady after 40 h, indicating the formation of SEI layer stable enough to resist oxygen and water corruption to the lithium anode could be formed by the facilitation of IL-TEMPO. It could partially explain the superior cycling stability of IL-TEMPO electrolyte than the DEGDME electrolyte.

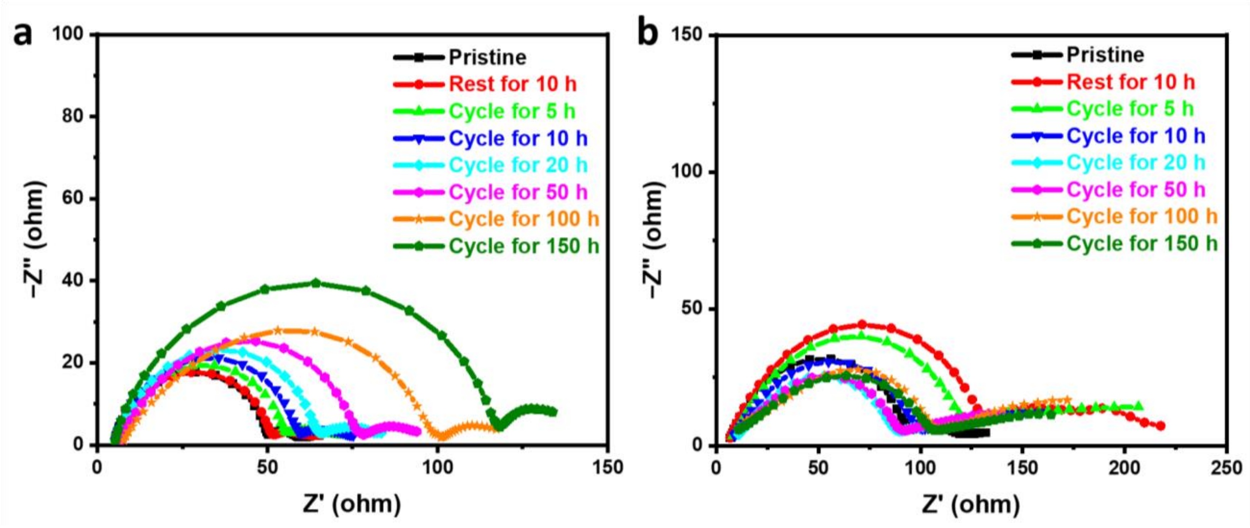

Supplementary Figure 27 | The lithium stability investigation in Li|Li symmetric cells in argon atmosphere during cycling. a. Impedance spectra of the symmetric cell with DEGDME electrolyte. **b.** Impedance spectra of the symmetric cell with IL-TEMPO (1 %) containing electrolyte.

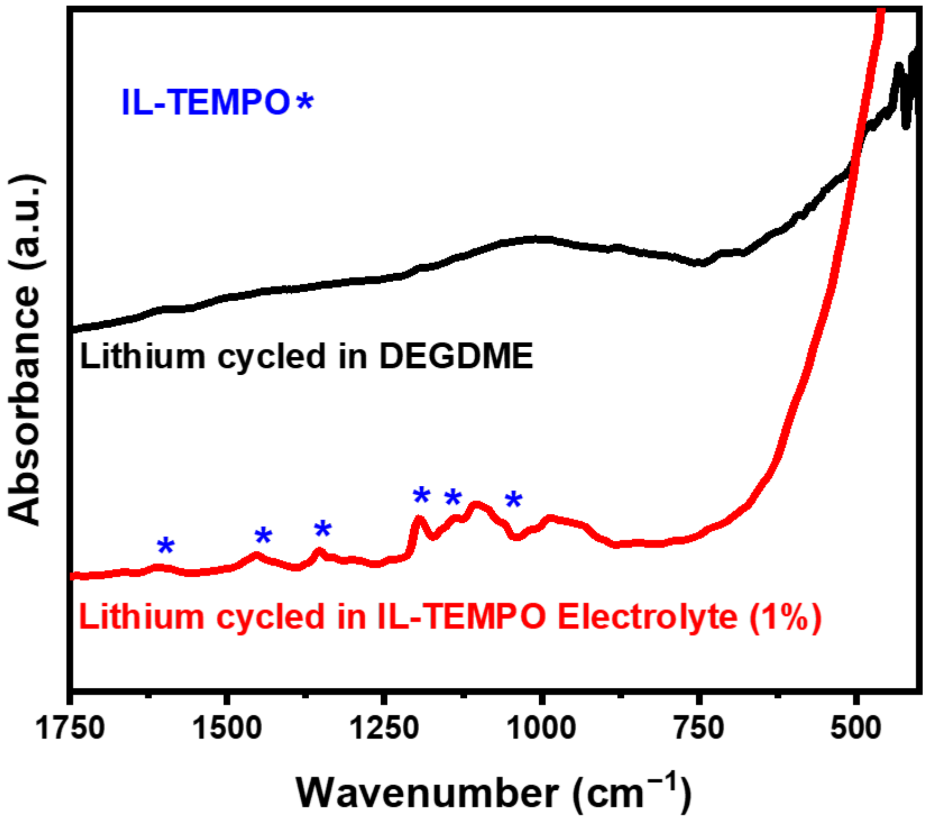

Supplementary Figure 28 | FTIR spectra of the lithium metals cycled in different electrolytes. The clearly presented characteristic peaks related to the TEMPO moiety demonstrates that IL-TEMPO is inserted in the SEI layer on the lithium anode.

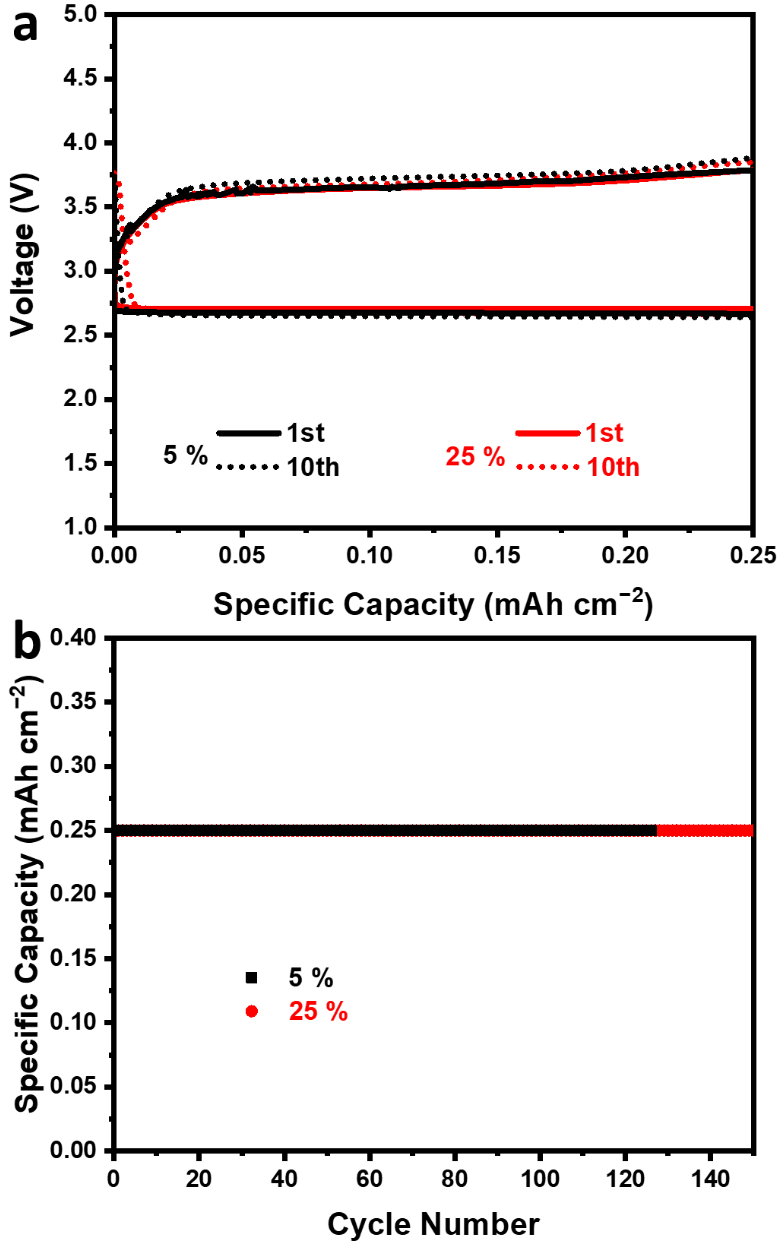

Supplementary Figure 29 | The electrochemical performances of the Li-O_2 batteries with IL-TEMPO (different concentration) contained electrolytes. a. The discharge-charge profiles of the Li-O_2 batteries with IL-TEMPO electrolyte (5 %) and IL-TEMPO electrolyte (25 %). **b.** The cycling performances of the Li-O_2 batteries. The concentrations of LiTFSI were both 0.5 M. The current densities were 0.1 mA cm^{-2} and the cut-off voltage was 2.3 V/4.6 V.

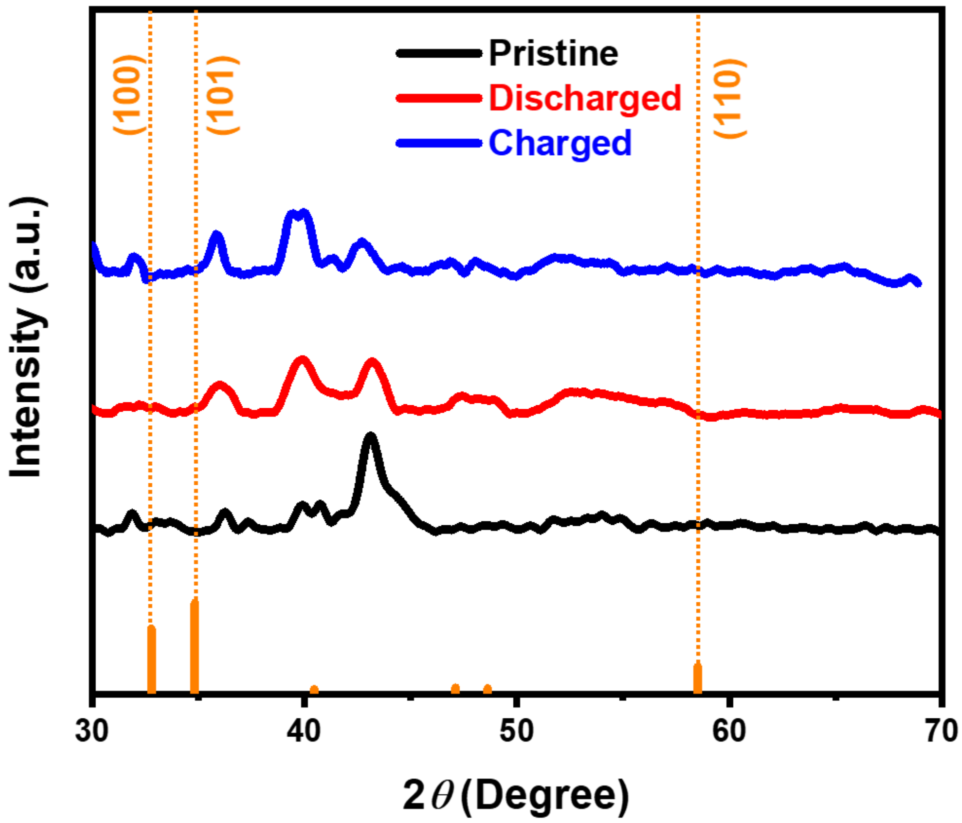

Supplementary Figure 30 | XRD spectra of the CNT electrodes before, during and after first cycle. The electrolyte used is IL-TEMPO (50 %). The spectra are indexed with standard Li_2O_2 peaks.

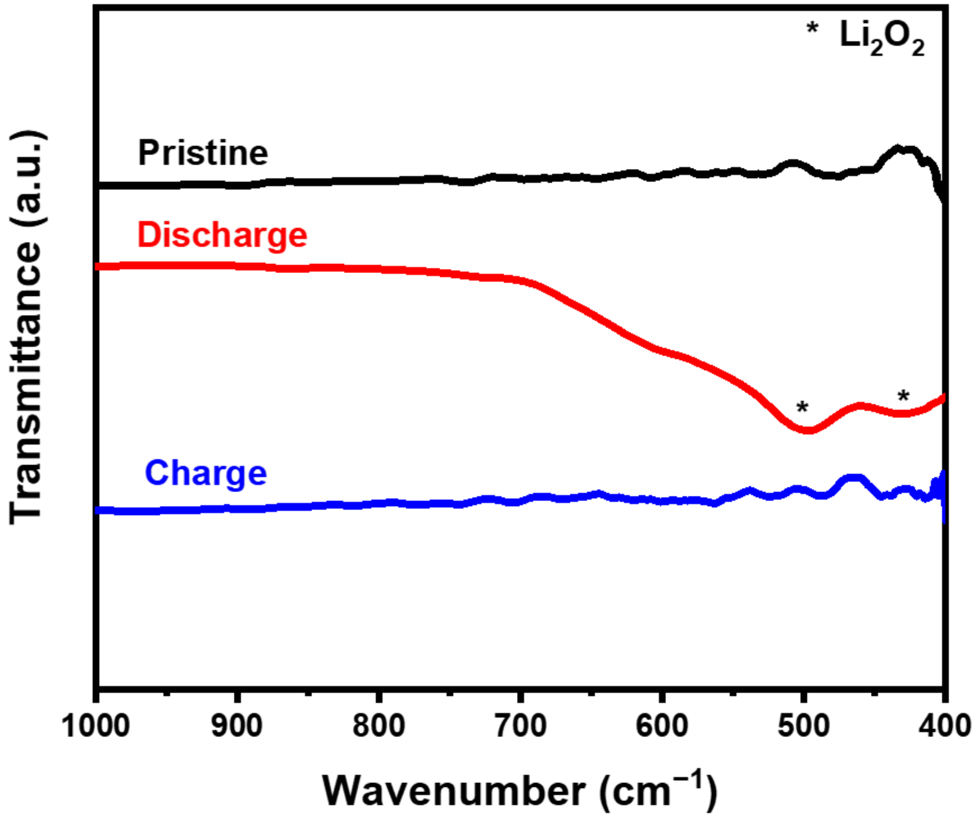

Supplementary Figure 31 | FTIR spectra of the electrode before and after cycling using the 50 % IL-TEMPO electrolyte.

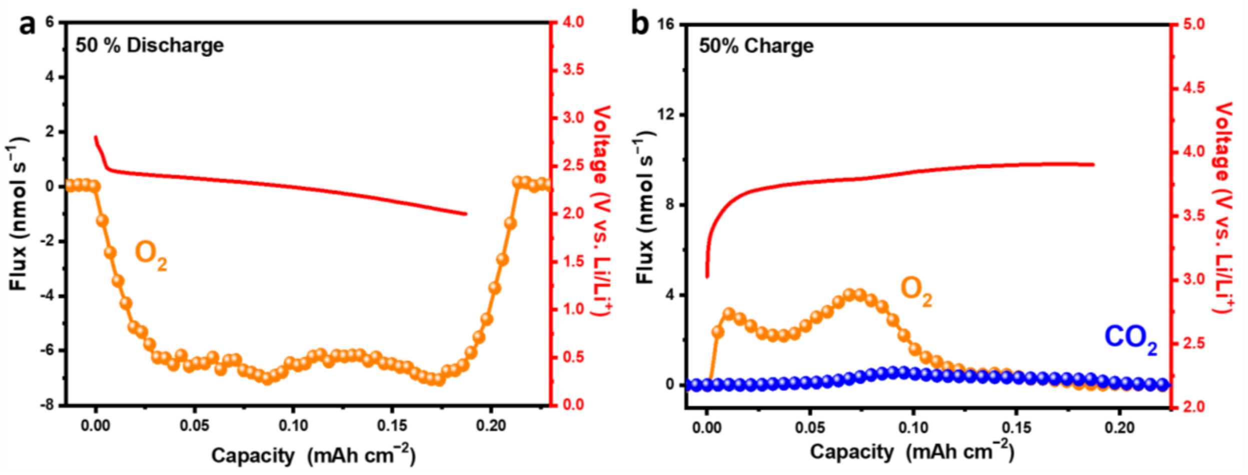

Supplementary Figure 32 | The mechanism illustration of the IL-TEMPO electrolyte (50 %) in a Li-O_2 cell. *In situ* DEMS analysis of the gas consumption and evolution during Li-O_2 cell operation: **a.** discharge to 2 V, and **b.** charge to the same discharge capacity. The current density was 0.25 mA cm^{-2}.

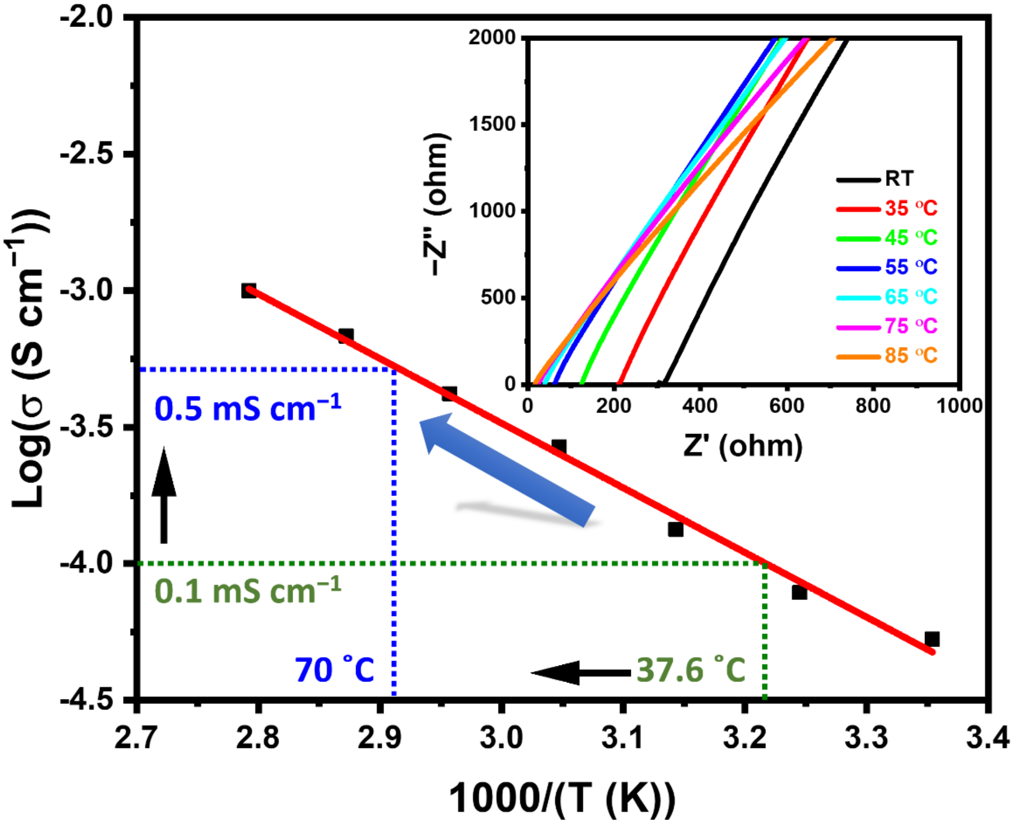

Supplementary Figure 33 | The ionic conductivity vs. temperature of the IL-TEMPO pure electrolyte. The concentration of LiTFSI was kept at 0.5 M. The cell was constructed by using a Stainless Steel (SS)|Electrolyte|SS configuration. The temperature was carefully controlled, and each scan of impedance was taken after resting at each certain temperature for 20 min. Based on the literature, electrolytes with ionic conductivity higher than 0.1 mS cm^{-1} are suitable used in lithium-based batteries[2]. We chose 70 ˚C as the ionic conductivity was 0.5 mS cm^{-1}, which was sufficient for Li-O_2 battery tests, providing acceptable electrochemical characteristics using relatively high current densities.

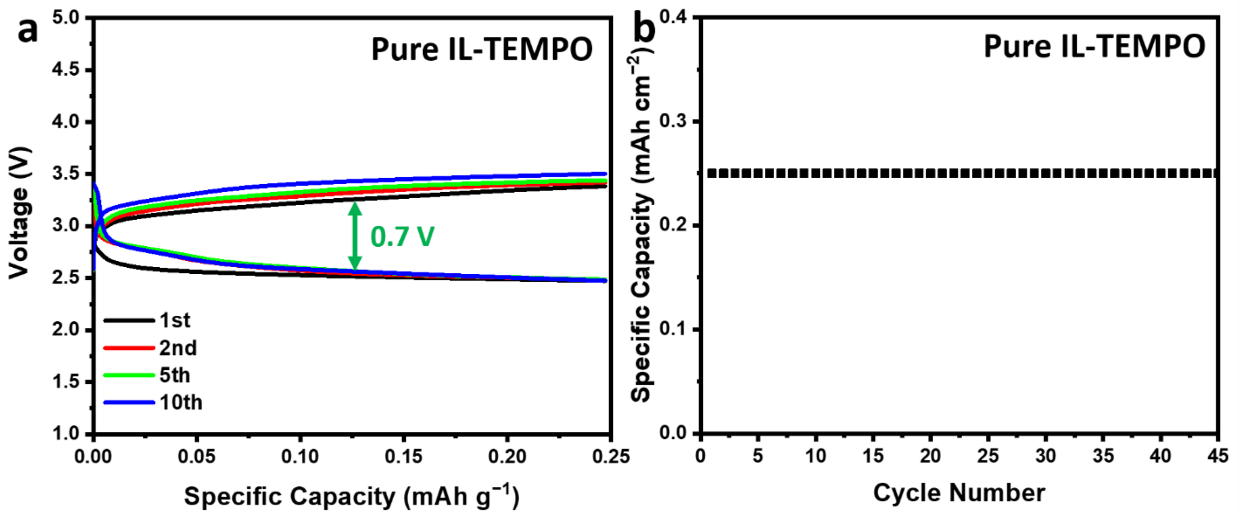

Supplementary Figure 34 | The electrochemical performances of the Li-O_2 battery with IL-TEMPO pure electrolyte. a. The discharge-charge profiles and **b.** the cycling performances of Li-O_2 battery with the pure IL-TEMPO electrolyte with no DEGDME solvent at 70 ^{o}C. The concentration of LiTFSI was 0.5 M, and the current density was 0.1 mA cm^{-2}. The charging over-potentials are further reduced compared with those when using 50 % ratio electrolyte and the cell performed smoothly at the high temperature, achieving a relatively long cycle life (Supplementary Figure 34b).

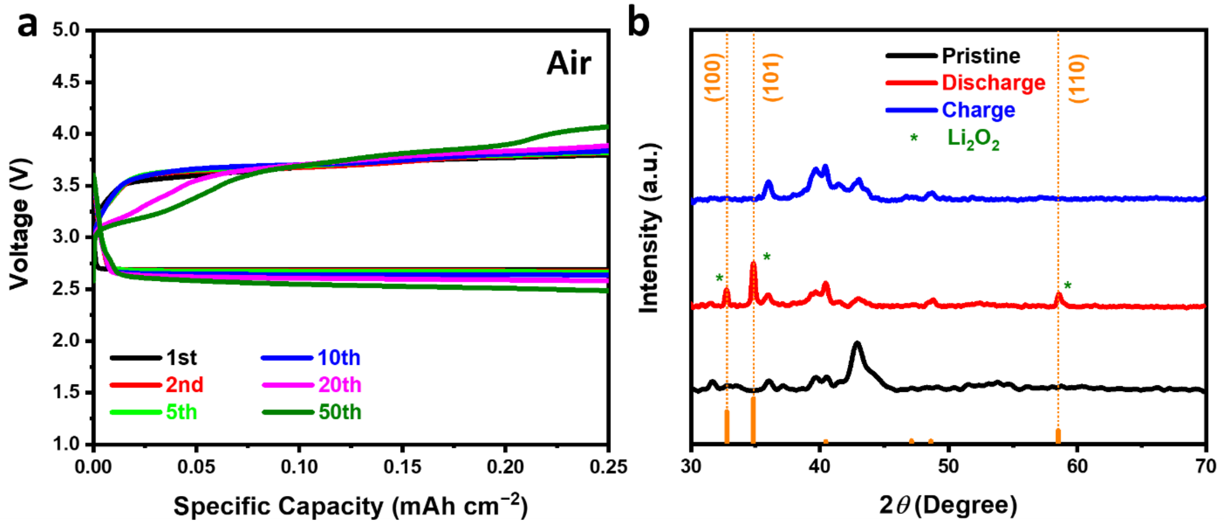

Supplementary Figure 35 | The electrochemical performance and post-mortem characterization of the Li-air batteries with 1 % IL-TEMPO electrolyte. **a.** The discharge-charge profiles of the Li-air battery. The concentration of LiTFSI was 0.5 M, and the current density was 0.1 mA cm^{-2}. **b.** The XRD spectra of the electrodes before discharge, after discharge, and after charge. The spectra are indexed with the standard Li_2O_2 peaks.

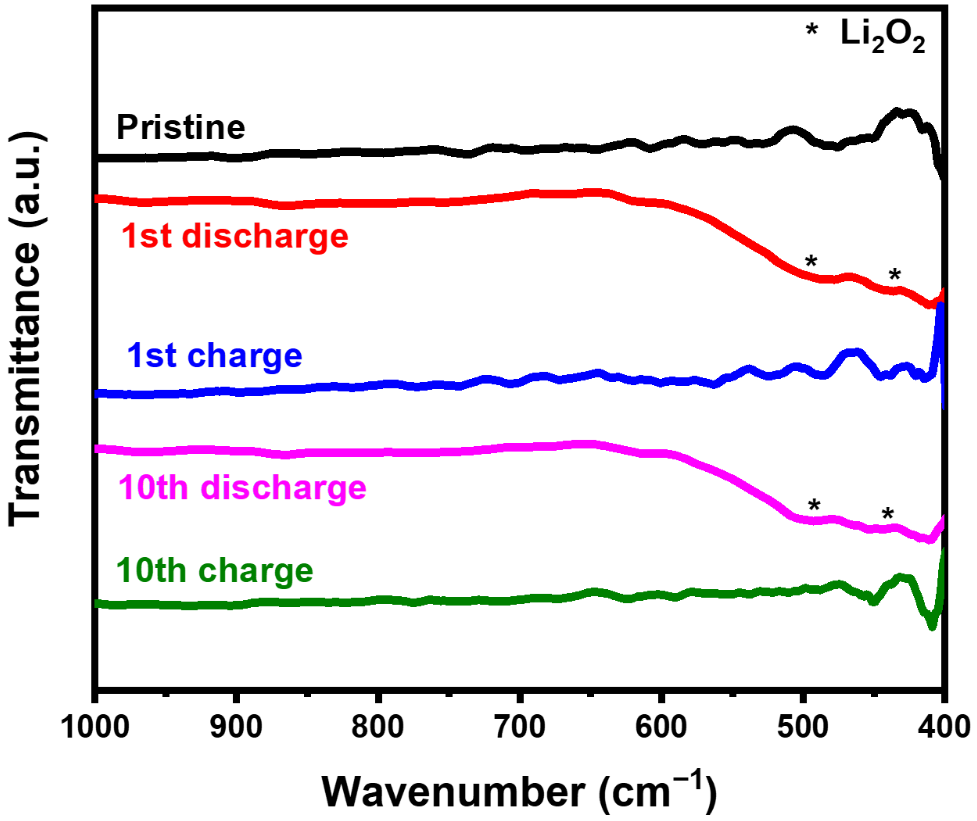

Supplementary Figure 36 | FTIR spectra of the electrodes before and after cycling using 1 % IL-TEMPO electrolyte in air atmosphere.

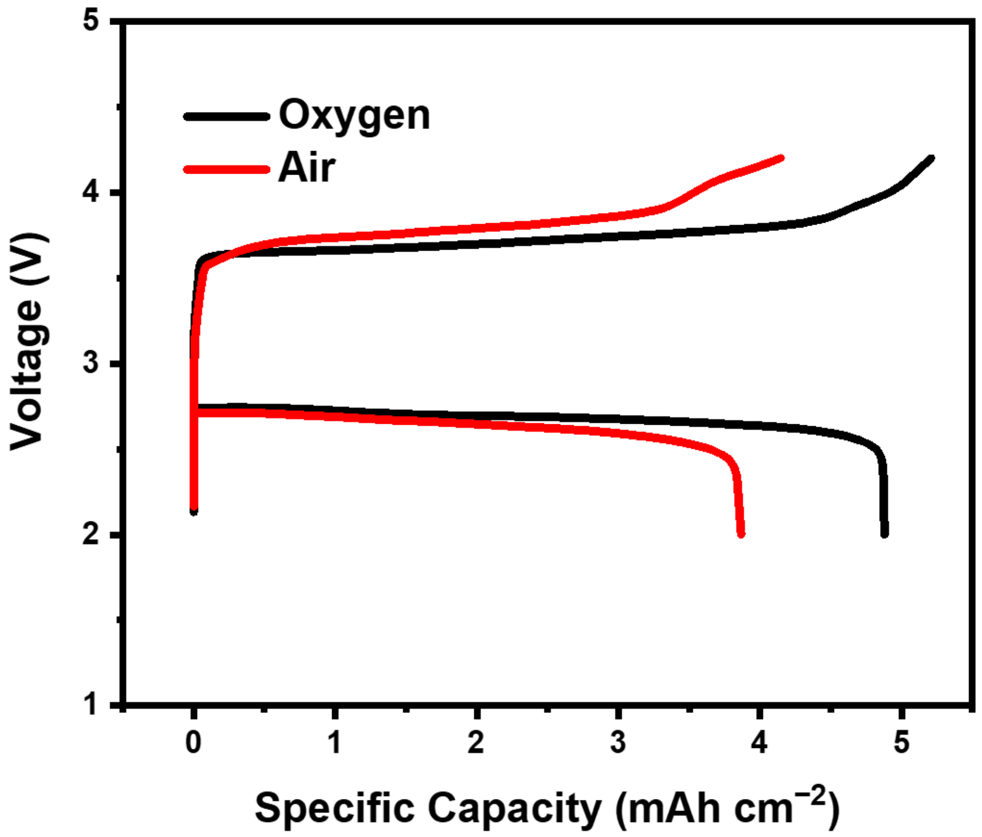

Supplementary Figure 37 | The full discharge-charge profiles of the Li-air/O_2 batteries with 1 % IL-TEMPO electrolyte. The concentration of LiTFSI was 0.5 M, and the current density was 0.1 mA cm^{-2}.

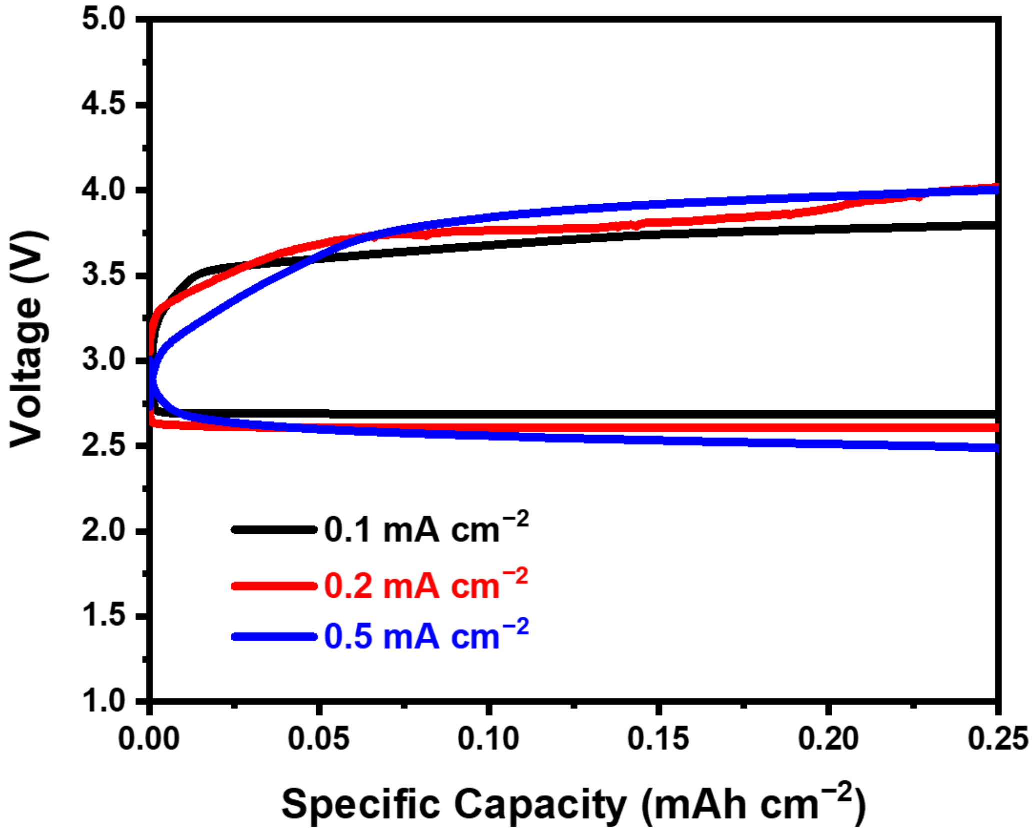

Supplementary Figure 38 | The discharge-charge profiles of the Li-air battery with 1 % IL-TEMPO electrolyte at different current densities.

Supplementary Tables

Supplementary Table 1 | Comparison of common solution-based redox shuttles used in Li-O_2 batteries

Additives	Discharge Capacity[a]	Charge Voltage	Anode[b]	Cycle[c]	Additional Role[d]	References
IL-TEMPO	3300%	3.6 V	Protective	200	Solvent	This work
TTF	N/A[e]	3.3 V-3.6 V	Corrosive	120	N/A	3-9
TTF LiCl	N/A	3.25 V	Protective	100	Organic Conductor	10
LiI	N/A	3.1 V-3.6 V	Corrosive	900	N/A	11-19
LiI	N/A	2.8 V (photoelectrode)	N/A	4	N/A	20
LiI GPE	N/A	3.6 V	Protective	400	Electrolyte	21
$LiI(HPN)_2$	N/A	3.6 V	N/A	N/A	N/A	22
KI	175%	3.5 V	N/A	23	N/A	23
CsI	N/A	3.5 V	Protective	125	N/A	24
InI_3	N/A	3.5 V	Protective	50	N/A	25
LiBr	N/A	3.5 V	N/A	40	Salt	26,27
$LiNO_3$	N/A	3.74 V	Protective	60	Salt	28,29
TEMPO	N/A	3.7 V	Corrosive	10	N/A	13,30-33
BHT	72%	3.2 V	N/A	N/A	N/A	34
MPT	N/A	3.63 V	Corrosive	50	N/A	35-37
TMPPA DTBBQ	N/A	3.7 V	N/A	3	N/A	38
TDPA	N/A	3.1 V, 3.5 V	N/A	100	N/A	39
Viologen	137.5%	N/A	N/A	N/A	N/A	40
Viologen LiI	N/A	3.55 V, 3.75 V	N/A	30	N/A	41

1-Me-AZADO	N/A	3.6 V	N/A	8	N/A	42
FePc	150%	3.6 V	N/A	140	N/A	43
DBBQ	4091%	-	-	-	-	44
DBBQ TEMPO	2000%	3.6 V	Corrosive	50	N/A	45
DBBQ TEMPO H_2O	1800%	3.65 V	N/A	20	N/A	46
DMPZ	N/A	3.1 V	Corrosive	45	N/A	47,48
DMPZ FEC	N/A	3.1 V	Protective	60	N/A	49
Heme	12%	3.1 – 4.0 V	N/A	55	N/A	50
Coenzyme-Q10	10000%	N/A	N/A	N/A	N/A	51
PTIO SDS	133%	3.6 V	Protective	150	N/A	52

a. The results are obtained by comparing the discharge capacities with the redox mediators to that without the redox mediators. The electrodes used in the references are different, but they are not mentioned in this table.

b. The anodes compared in the table are lithium anodes. The alternative anodes are not listed here. Also, the results here only represent the effects of the solution-based catalysts towards lithium anode, excluding the effects of external protection layers.

c. The numbers displayed in the table is the best cycle numbers among the chosen references. The uses of external artificial separators or protection layers are also involved, though not clearly mentioned in this table.

d. Additional role means that the redox shuttles may play other critical roles in the $Li-O_2$ batteries except protecting lithium anode and facilitating discharge and charge processes.

e. N/A in this chart means either not mentioned in the references, or not suitable for facilitating certain processes.

Supplementary Table 2 | Discharge Li_2O_2 percent yield (Y_{Li2O2}) for the cells under O_2 and air atmosphere

Electrolyte	Atmosphere	Capacity (mAh)	Li_2O_2 (μmol)	Y_{Li2O2} (%)
1 % IL-TEMPO	O_2	2.08±0.02	36±1	92.7±1.7
	Air	2.08±0.02	32±2	82.6±4.5
50 % IL-TEMPO	O_2	1.10±0.02	13±5	63.2±24.1
	Air	1.10±0.02	14±3	67.5±13.4

The error is the deviation from three replicate trials.

Supplementary Table 3 | DEMS results of Li-O_2 batteries with different electrolytes

	Charge passed[a]		O_2 quantity		e^-/O_2		CO_2 quantity	
	D[b]	R[b]	C[b]	E[b]	D[b]	R[b]	C[b]	E[b]
**DEGDME electrolyte**	18.65	17.89	9.01±0.45	8.43±0.25	2.07	2.12	-	0.464
**IL-TEMPO electrolyte (1%)**	18.64	18.64	9.18±0.45	9.49±0.28	2.03	1.96	-	0.134

a. The current density of discharge process is 0.25 mA cm^{-2}, and the current density of charge process is 0.125 mA cm^{-2}.

b. μmol/mg, the quantities of charge and gas have been normalized to the total mass of the respective cathodes. D:discharge; R:recharge; C:consumption of gas during discharge; E:evolution of gas during recharge.

c. The error during discharge is considered as 5 %, and during charge is considered as 3 %.

Supplementary Notes

Supplementary Note 1: The potential for the reduction of the N-O radical undergoes a positive shift after the first cycle as shown in Supplementary Figure 3a, which could be due to the formation of a solid electrolyte interface (SEI) on the surface of the lithium anode. This has been proven by monitoring the a.c. impedance spectra change after each cycles in Supplementary Figure 3b. Meanwhile, the CV profiles in the second and third cycles (Supplementary Figure 3a) match perfectly with the CV curves obtained using pre-treated lithium anode (Figure 1b), further confirming the SEI formation during the CV tests.

Supplementary Note 2: The possible cause for the slight differences between Supplementary Figure 5 and 6 are the stainless steel current collector does not have the same porous structure as the carbon paper. Therefore, the IL-TEMPO molecules could easily diffused away from the electrode, resulting in the slightly decrease of the reversible peaks corresponding to the reduction/oxidation of IL-TEMPO.

Supplementary Note 3: DMA is used as it has higher oxygen solubility. The image clearly shows that when contact with the crystal and metal surface, large quantity of bubble was released. This experiment indicates that oxygen can interact with chemically reduced IL-TEMPO to form highly unstable intermediate, which cannot be directly detected by FTIR (Supplementary Figure 9c) due to the immediate decomposition when contact with metal surface. The detailed information is shown in the supporting video.

Supplementary Note 4: The charge capacity is slightly higher than the discharge capacity until an expected increase of over-potential is observed. The additional capacity is contributed by the self-redox of the IL-TEMPO which has similar value as in Supplementary Figure 15a. The consequent discharge curve shows additional capacity before the beginning of the Li_2O_2 formation, which is directly related to the reduction of the accumulated oxidized IL-TEMPO during the overcharge process. The distinctively different discharge curves in the non-overcharge curves

proves that the contribution of the self-redox of IL-TEMPO is negligible in the normal operation of Li-O_2 batteries.

Supplementary Note 5: The extension of the capacity could potentially increase the possibility of side-reactions between the metastable Li_2O_2 and the DEGDME electrolyte/carbon electrode due to the extended exposing time. Furthermore, the large quantity of discharge products may cause the expansion of CNT electrode, which could lead to the loss of contact between the electrode materials, thus negatively affected the cycle life of Li-O_2 cells. Nevertheless, the Li-O_2 cells still maintain exceptional cycling performance with the capacity limitations extended to 1 mAh cm^{-2} and 2 mAh cm^{-2}, indicating the exceptional capability of IL-TEMPO to increase the cycling performance of Li-O_2 batteries.

Supplementary Note 6: The samples were prepared by soaking the electrolyte-adsorbed glass fibre separator in 3 ml DEGDME solvent, and the solutions were directly used for UV tests. The UV spectrum of the electrolyte after the first cycle shows that the concentration of IL-TEMPO slightly reduces, due to the incorporation of small amount of IL-TEMPO during the formation of SEI on the lithium anode. However, the concentration of IL-TEMPO stabilizes during the following cycles. The concentration after 50 cycles is almost the same as the one obtained after the first cycle. There is no additional peaks observed in the UV spectra. Furthermore, the FTIR spectra of the electrolytes remain unchanged after 50 cycles. This clearly indicates that there is almost negligible side-reactions occurred during the battery reactions in the 50 cycles.

Supplementary Note 7: The impedance slightly increases when a DEGDME electrolyte was used (Supplementary Figure 25a), which is probably related to the facts that DEGDME solvent is not capable of forming solid electrolyte interface (SEI) and the formation of SEI mainly originates from the decomposition of LiTFSI facilitated by lithium metal. However, in Supplementary Figure 25b, the impedance of the close cell with IL-TEMPO significantly increases in the first 25 h and stabilizes since then. The proposed mechanism is that IL-TEMPO would facilitate the formation

of SEI layer on the surface of lithium metal in the first few hours, and the SEI layer would prevent further reactions by physically separates the components. This phenomenon is particularly important as the SEI layer could prevent the side-reactions caused by redox mediators and oxygen in the electrolyte.

Supplementary Note 8: The impedance of the cell with IL-TEMPO increases in the first 10 h rest due to the formation of SEI layer, and decreases during cycling. This phenomenon indicates that the insertion of IL-TEMPO molecules can be accelerated during the electrochemical process, and the existence of IL-TEMPO can significantly enhance the ion transport through the SEI layer, which can be seen by comparing the impedance spectra between in Supplementary Figure 27a and b.

Supplementary Note 9: Oxygen was continuously consumed during discharge (e^{-}/O_2 = 1.96) which is similar to the electrolyte with low concentration of IL-TEMPO. However, the charge behavior significantly changed, as the amount of oxygen emitted significantly reduced during the DEMS operation and the charging over-potential slightly increased when oxygen evolution stopped. Additionally, there are two evolution peaks shown in Supplementary Figure 32b which may be originated from two different discharge products (solution-based intermediates and solid-based amorphous Li_2O_2). The phenomenon is probably related to the possibility that during the degassing process for DEMS test, the majority of highly unstable intermediates which were formed during discharge process decomposed during the gas flow. Therefore, the amount of oxygen emitted would be significantly reduced in the following test, and the second plateau with slightly increased over-potential was caused by the self-redox reaction when discharge products were all consumed. It should be noted that there should not be any forms of Li_2O_2 remaining when self-redox reaction occurs, because the oxidized form of IL-TEMPO would react with Li_2O_2 to emit O_2 which can be detected by DEMS (proven in Figure 3f), further demonstrating the assertion of oxygen loss during degassing process.

Supplementary Method

Synthesis of IL-TEMPO

The detailed synthesis process of 1,2-dimethyl-3-(4-(2,2,6,6-tetramethyl-1-oxyl-4-piperidoxyl)-pentyl)imidazolium bis(trifluoromethane)sulfonimide (IL-TEMPO) is illustrated in Supplementary Figure 1a[53-56]. In brief, 10 mmol 2,2,6,6-tetramethyl-4-piperidinol (95%, Sigma-Aldrich) was dissolved in methanal at room temperature together with 1 mmol $Na_2WO_4 \cdot 2H_2O$ (≥99%, Sigma-Aldrich) and 5 mL 30% H_2O_2 (Chem-suppy). The mixture was stirred at 60 ^oC for 24 h under reflux. After the reaction, the solvents were eliminated through rotavapor (Buchi R-100) and chlorobenzene (99.8%, Sigma-Aldrich) was added to dissolve the precipitation. After filtration, the filtrate was collected and dried under vacuum at room temperature. The obtained red solid was 4-hydroxy-TEMPO.

The obtained 4-hydroxy-TEMPO (5 mmol) was dissolved in anhydrous acetone (pre-dried, Sigma-Aldrich) with the slow addition of 10 mmol NaH (90%, Sigma-Aldrich) at 0 ^oC. The reaction was kept for 15 min when 12 mmol 1,5-dibromopentane (97%, Sigma-Aldrich) was added to the mixture which was remained for 3 h. The solvent was removed under vacuum and 30 mL water was added to dissolve the mixture. The aqueous layer was extracted using methylene chloride (Chem-supply), which was concentrated under vacuum. The resultant red liquid was then purified by chromatography (hexane and methylene chloride with different ratio were used, Chem-supply) to obtain viscous red liquid, which was 5-TEMPO-pentyl bromide.

Further reaction between 5-TEMPO-pentyl bromide (3 mmol) and 1,2-dimethylimidazole (3.5 mmol, 98%, Sigm-Aldrich) was conducted in 10 mL acetonitrile (99.8%, Sigma-Aldrich) at 60 ^oC for 24 h. After cooling down to room temperature, 50 mL diethyl ether (Chem-supply) was added to cause precipitation. The precipitation was washed with diethyl ether and dried under vacuum to obtain 1,2-dimethyl-3-(5-TEMPO-pentyl) imidazolium bromide.

^1H NMR (DMSO-d_6, ppm): d = 1.04 (s, 6H, CH_3), 1.07 (s, 6H, CH_3), 1.19–1.34 (m, 4H, $C(3)_{Pip}$-H and $C(5)_{Pip}$-H, -CH_2-CH_2-CH_2-N<), 1.46–1.52 (m, 2H, -CH_2-CH_2-N<), 1.68–1.76 (m, 2H, -O-CH_2-CH_2-), 1.79–1.86 (m, 2H, $C(3)_{Pip}$-H and $C(5)_{Pip}$-H), 2.58 (s, 3H, $C(2)_{Im}$-CH_3), 3.36 (t, 2H, $^3J(H,H)$ = 6.5 Hz, -O-CH_2-), 3.45–3.54 (m, 1H, $C(4)_{Pip}$-H), 3.75 (s, 3H, $N(1)_{Im}$-CH_3), 4.10 (t, 2H,

^3J(H,H) = 7.3 Hz, $-CH_2-N\leq$), 7.62–7.65 (m, 1H, $C(4)_{Im}$-H or $C(5)_{Im}$-H), 7.65–7.67 (m, 1H, $C(4)_{Im}$-H or $C(5)_{Im}$-H).

Further ion exchange process was operated in water with the addition of lithium bis(trifluoromethane)sulfonimide (LiTFSI, 99.95%, Sigma-Aldrich), which resulted in a viscous oil-like red ionic liquid (IL-TEMPO) which is immiscible with water. (Anal. Calcd for $C_{21}H_{35}F_6N_4O_6S_2 \cdot 1.5H_2O$: C 39.12, H 5.94, N 8.69. Found: C 39.42, H 5.74, N 8.26) The ionic liquid was washed with water and dried at 100 ^oC under vacuum for 24 h before use.

^{13}C NMR (DMSO-d_6, ppm): d = 9.18 ($C(2)_{Im}-CH_3$), 20.83 ($(CH_3)_{Pip}$), 22.76 ($-CH_2-(CH_2)_2-N<$), 29.19 ($-CH_2-CH_2-N<$), 29.34 ($-O-CH_2-CH_2-$), 32.60 ($(CH_3)_{Pip}$), 34.75 ($N(1)_{Im}-CH_3$), 44.93 ($C(3)_{Pip}$, $C(5)_{Pip}$), 47.70($-CH_2-N\leq$), 58.19 ($C(2)_{Pip}$, $C(6)_{Pip}$), 67.19 ($-O-CH_2-$), 70.31 ($C(4)_{Pip}$), 119.82 (q, CF_3, ^1J(C,F) = 322 Hz), 120.96, 122.43 ($C(4)_{Im}$, $C(5)_{Im}$), 144.12 ($C(2)_{Im}$).

Demonstration experiments

The demonstration experiments of the catalytic capability of IL-TEMPO towards Li_2O_2 and Li_2CO_3 were conducted by directly charging the cells with electrodes loaded with Li_2O_2 or Li_2CO_3. The electrodes were prepared by paste the mixture of CNT, PVDF and Li_2O_2/Li_2CO_3 (4:1:4) in NMP on the surface of the pre-cut glass fibre. All the preparation process is done in an Ar-filled glovebox. The electrodes were dried under vacuum before use. The cells were assembled by using lithium metal foil as anode, IL-TEMPO electrolyte (1 %) in glass fibre separator, and the as-prepared electrodes as cathode. The cut-off voltage was 4.6 V, and current density was 0.1 mA cm^{-2}.

The demonstration experiments of interactions between IL-TEMPO and oxygen were conducted by altering the electrochemical reduction of IL-TEMPO into chemical reduction with the aid of phenylhydrazine ($PhNHNH_2$) and N,N-dimethylacetamide (DMA). Four solutions were preparing with the formula of $PhNHNH_2$, DMA+LiTFSI, DMA+LiTFSI+IL-TEMPO, and DMA+LiTFSI+IL-TEMPO+$PhNHNH_2$. ATR-FTIR was performed to the solution before and after purging with oxygen for 5 min.

Li_2O_2 titration method

The yields of Li_2O_2 after the discharge process of Li-O_2 batteries are measured through a Li_2O_2 titration method which is reported previously in the literature[57]. Typically, the electrodes are extracted from the discharged cells and dried under vacuum in the glovebox. The electrodes are then transferred outside the glovebox in a sealed bottle, where 2 mL of ultrapure water (18.2 MΩ cm, Millipore) is injected. The contents in the bottle are vigorously stirred for 30 seconds and a drop of phenolphalein in isopropanol is added as the end-point indicator. The contents are titrated with a 0.005M HCl solution. The main reaction is shown below.

$$Li_2O_2 + 2H_2O \rightarrow 2LiOH + H_2O_2 \qquad \text{(Equation 1)}$$

A mixure of 1mL 2 wt% KI in H_2O, 1mL 3.5 M H_2SO_4, and 50 µL molybdate-based catalyst solution (1g $(NH_4)_2MoO_4$ in 10 mL 6N NH_3H_2O with 3g NH_4NO_3, which is then diluted to 50 mL using ultrapure H_2O) is added in the bottle, which turns yellow due to the formation of I_2.

$$H_2O_2 + 2KI + H_2SO_4 \rightarrow I_2 + K_2SO_4 + 2H_2O \qquad \text{(Equation 2)}$$

The as-formed I_2 is immediately titrated by 0.01 N NaS_2O_3 to a faint straw colour, when 0.5 mL 1 % starch indicator is added to turn the solution blue. The titration is continued until the solution turns clear.

$$I_2 + 2Na_2S_2O_3 \rightarrow Na_2S_4O_6 + 2NaI \qquad \text{(Equation 3)}$$

Supplementary References

1. Guo, W., Yin, Y.-X., Xin, S., Guo, Y.-G. & Wan, L.-J. Superior radical polymer cathode material with a two-electron process redox reaction promoted by graphene. *Energy Environ. Sci.* **5**, 5221−5225 (2012).
2. Li, Q., Chen, J., Fan, L., Kong, X. & Lu, Y. Progress in electrolytes for rechargeable Li-based batteries and beyond. *Green Energy Environ.* **1**, 18−42 (2016).
3. Torres, W. R., Herrera, S. E., Tesio, A. Y., del Pozo, M. & Calvo, E. J. Soluble TTF catalyst for the oxidation of cathode products in Li-oxygen battery: A chemical scavenger. *Electrochim. Acta* **182**, 1118−1123 (2015).
4. Qiao, Y. & Ye, S. Spectroscopic investigation for oxygen reduction and evolution reactions with tetrathiafulvalene as a redox mediator in Li-O_2 battery. *J. Phys. Chem. C* **120**, 15830−15845 (2016).
5. Qiao, Y. *et al.* MOF-based separator in an Li-O_2 battery: An effective strategy to restrain the shuttling of dual redox mediators. *ACS Energy Lett.* **3**, 463−468 (2018).
6. Schaltin, S., Vanhoutte, G., Wu, M., Bardé, F. & Fransaer, J. A QCM study of ORR-OER and an in situ study of a redox mediator in DMSO for Li-O_2 batteries. *Phys. Chem. Chem. Phys.* **17**, 12575−12586 (2015).
7. Han, J. *et al.* Full performance nanoporous graphene based Li-O_2 batteries through solution phase oxygen reduction and redox-additive mediated Li_2O_2 oxidation. *Adv. Energy Mater.* **7**, 1601933 (2017).
8. Chen, Y., Freunberger, S. A., Peng, Z., Fontaine, O. & Bruce, P. G. Charging a Li-O_2 battery using a redox mediator. *Nat. Chem.* **5**, 489 (2013).
9. Yang, C. *et al.* Direct observations of the formation and redox-mediator-assisted decomposition of Li_2O_2 in a liquid-cell Li-O_2 microbattery by scanning transmission electron microscopy. *Adv. Mater.* **29**, 1702752 (2017).
10. Zhang, J., Sun, B., Zhao, Y., Kretschmer, K. & Wang, G. Modified tetrathiafulvalene as an organic conductor for improving performances of Li-O_2 batteries. *Angew. Chem. Int. Ed.* **56**, 8505−8509 (2017).
11. Togasaki, N., Shibamura, R., Naruse, T., Momma, T. & Osaka, T. Prevention of redox shuttle using electropolymerized polypyrrole film in a lithium-oxygen battery. *APL Mater.* **6**, 047704 (2018).
12. Landa-Medrano, I. *et al.* Operando UV-visible spectroscopy evidence of the reactions of iodide as redox mediator in Li-O_2 batteries. *Electrochem. Commun.* **59**, 24−27 (2015).
13. Zhang, W. *et al.* Promoting Li_2O_2 oxidation via solvent-assisted redox shuttle process for low overpotential Li-O_2 battery. *Nano Energy* **30**, 43−51 (2016).
14. Nakanishi, A. *et al.* Electrolyte composition in Li/O_2 batteries with LiI redox mediators: solvation effects on redox potentials and implications for redox shuttling. *J. Phys. Chem. C* **122**, 1522−1534 (2018).
15. Burke, C. M. *et al.* Implications of 4 e-oxygen reduction via iodide redox mediation in Li-O_2 batteries. *ACS Energy Lett.* **1**, 747−756 (2016).
16. Kwak, W.-J. *et al.* Understanding the behavior of Li-oxygen cells containing LiI. *J. Mater. Chem. A* **3**, 8855−8864 (2015).
17. Tułodziecki, M. *et al.* The role of iodide in the formation of lithium hydroxide in lithium-oxygen batteries. *Energy Environ. Sci.* **10**, 1828−1842 (2017).

18. Lim, H. D. *et al.* Superior rechargeability and efficiency of lithium-oxygen batteries: hierarchical air electrode architecture combined with a soluble catalyst. *Angew. Chem. Int. Ed.* **53**, 3926−3931 (2014).
19. Zhu, Y. G. *et al.* Proton enhanced dynamic battery chemistry for aprotic lithium-oxygen batteries. *Nat. Commun.* **8**, 14308 (2017).
20. Yu, M., Ren, X., Ma, L. & Wu, Y. Integrating a redox-coupled dye-sensitized photoelectrode into a lithium-oxygen battery for photoassisted charging. *Nat. Commun.* **5**, 5111 (2014).
21. Guo, Z., Li, C., Liu, J., Wang, Y. & Xia, Y. A long-life lithium-air battery in ambient air with a polymer electrolyte containing a redox mediator. *Angew. Chem.* **129**, 7613−7617 (2017).
22. Li, Y. *et al.* Li-O_2 cell with LiI(3-hydroxypropionitrile)$_2$ as a redox mediator: Insight into the working mechanism of I^- during charge in anhydrous systems. *J. Phys. Chem. Lett.* **8**, 4218−4225 (2017).
23. Landa-Medrano, I. *et al.* Potassium salts as electrolyte additives in lithium-oxygen batteries. *J. Phys. Chem. C* **121**, 3822−3829 (2017).
24. Lee, C. K. & Park, Y. J. CsI as multifunctional redox mediator for enhanced Li–air batteries. *ACS Appli. Mater. Inter.* **8**, 8561−8567 (2016).
25. Zhang, T., Liao, K., He, P. & Zhou, H. A self-defense redox mediator for efficient lithium-O_2 batteries. *Energy Environ. Sci.* **9**, 1024−1030 (2016).
26. Kwak, W.-J. *et al.* Li-O_2 cells with LiBr as an electrolyte and a redox mediator. *Energy Environ. Sci.* **9**, 2334−2345 (2016).
27. Liang, Z. & Lu, Y.-C. Critical role of redox mediator in suppressing charging instabilities of lithium-oxygen batteries. *J. Am. Chem. Soc.* **138**, 7574−7583 (2016).
28. Lim, H.-D. *et al.* Three-dimensionally branched carbon nanowebs as air-cathode for redox-mediated Li-O_2 batteries. *Carbon* **118**, 114−119 (2017).
29. Sun, B., Huang, X., Chen, S., Zhang, J. & Wang, G. An optimized $LiNO_3$/DMSO electrolyte for high-performance rechargeable Li-O_2 batteries. *RSC Adv.* **4**, 11115−11120 (2014).
30. Bergner, B. J. *et al.* How to improve capacity and cycling stability for next generation Li-O_2 batteries: Approach with a solid electrolyte and elevated redox mediator concentrations. *ACS Appli. Mater. Inter.* **8**, 7756−7765 (2016).
31. Bergner, B. J., Schürmann, A., Peppler, K., Garsuch, A. & Janek, J. r. TEMPO: a mobile catalyst for rechargeable Li-O_2 batteries. *J. Am. Chem. Soc.* **136**, 15054−15064 (2014).
32. Lee, D. J., Lee, H., Kim, Y. J., Park, J. K. & Kim, H. T. Sustainable redox mediation for lithium-oxygen batteries by a composite protective layer on the lithium-metal anode. *Adv. Mater.* **28**, 857−863 (2016).
33. Chen, Y., Gao, X., Johnson, L. R. & Bruce, P. G. Kinetics of lithium peroxide oxidation by redox mediators and consequences for the lithium-oxygen cell. *Nat. Commun.* **9**, 767 (2018).
34. Yu, W. *et al.* A soluble phenolic mediator contributing to enhanced discharge capacity and low charge overpotential for lithium-oxygen batteries. *Electrochem. Commun.* **79**, 68−72 (2017).
35. Feng, N., Mu, X., Zhang, X., He, P. & Zhou, H. Intensive study on the catalytical behavior of N-Methylphenothiazine as a soluble mediator to oxidize the Li_2O_2 cathode of the Li-O_2 battery. *ACS Appli. Mater. Inter.* **9**, 3733−3739 (2017).

36. Ha, S. *et al.* Investigation into the stability of Li metal anodes in Li-O_2 batteries with a redox mediator. *J. Mater. Chem. A* **5**, 10609−10621 (2017).
37. Zhu, Y. G., Wang, X., Jia, C., Yang, J. & Wang, Q. Redox-mediated ORR and OER reactions: redox flow lithium oxygen batteries enabled with a pair of soluble redox catalysts. *ACS Catal.* **6**, 6191−6197 (2016).
38. Feng, N., He, P. & Zhou, H. Enabling catalytic oxidation of Li_2O_2 at the liquid-solid interface: the evolution of an aprotic Li-O_2 battery. *ChemSusChem* **8**, 600−602 (2015).
39. Kundu, D., Black, R., Adams, B. & Nazar, L. F. A highly active low voltage redox mediator for enhanced rechargeability of lithium-oxygen batteries. *ACS Cent. Sci.* **1**, 510−515 (2015).
40. Yang, L., Frith, J., Garcia-Araez, N. & Owen, J. R. A new method to prevent degradation of lithium-oxygen batteries: reduction of superoxide by viologen. *Chem. Commun.* **51**, 1705−1708 (2015).
41. Zhu, Y. G. *et al.* Dual redox catalysts for oxygen reduction and evolution reactions: towards a redox flow Li-O_2 battery. *Chem. Commun.* **51**, 9451−9454 (2015).
42. Bergner, B. J. *et al.* Understanding the fundamentals of redox mediators in Li-O_2 batteries: a case study on nitroxides. *Phys. Chem. Chem. Phys.* **17**, 31769−31779 (2015).
43. Sun, D. *et al.* A solution-phase bifunctional catalyst for lithium-oxygen batteries. *J. Am. Chem. Soc.* **136**, 8941−8946 (2014).
44. Gao, X., Chen, Y., Johnson, L. & Bruce, P. G. Promoting solution phase discharge in Li-O_2 batteries containing weakly solvating electrolyte solutions. *Nat. Mater.* **15**, 882 (2016).
45. Gao, X., Chen, Y., Johnson, L. R., Jovanov, Z. P. & Bruce, P. G. A rechargeable lithium-oxygen battery with dual mediators stabilizing the carbon cathode. *Nat. Energy* **2**, 17118 (2017).
46. Liu, T. *et al.* The effect of water on quinone redox mediators in nonaqueous Li-O_2 batteries. *J. Am. Chem. Soc.* **140**, 1428−1437 (2018).
47. Lee, S. H., Park, J. B., Lim, H. S. & Sun, Y. K. An advanced separator for Li-O_2 batteries: maximizing the effect of redox mediators. *Adv. Energy Mater.* **7**, 1602417 (2017).
48. Lim, H.-D. *et al.* Rational design of redox mediators for advanced Li-O_2 batteries. *Nat. Energy* **1**, 16066 (2016).
49. Kwak, W. J., Jung, H. G., Aurbach, D. & Sun, Y. K. Optimized bicompartment two solution cells for effective and stable operation of Li-O_2 batteries. *Adv. Energy Mater.* **7**, 1701232 (2017).
50. Ryu, W.-H. *et al.* Heme biomolecule as redox mediator and oxygen shuttle for efficient charging of lithium-oxygen batteries. *Nat. Commun.* **7**, 12925 (2016).
51. Zhang, Y. *et al.* High-capacity and high-rate discharging of a coenzyme Q10-catalyzed Li-O_2 battery. *Adv. Mater.* **30**, 1705571 (2018).
52. Xu, C. *et al.* Bifunctional redox mediator supported by an anionic surfactant for long-cycle Li-O_2 batteries. *ACS Energy Lett.* **2**, 2659−2666 (2017).
53. Lee, S. H. *et al.* Electrochemical properties of new organic radical materials for lithium secondary batteries. *J. Power Sources* **184**, 503−507 (2008).
54. Qian, W., Jin, E., Bao, W. & Zhang, Y. Clean and selective oxidation of alcohols catalyzed by ion-supported TEMPO in water. *Tetrahedron* **62**, 556−562 (2006).
55. Zhang, J. *et al.* A multi-functional gel co-polymer bridging liquid electrolyte and solid cathode nanoparticles: An efficient route to Li-O_2 batteries with improved performance. *Energy Storage Mater.* **7**, 1−7 (2017).

56. Zhang, J., Sun, B., Xie, X., Zhao, Y. & Wang, G. A Bifunctional organic redox catalyst for rechargeable lithium-oxygen batteries with enhanced performances. *Adv. Sci.* **3**, 1500285 (2016).
57. McCloskey, B. D. et al. Combining accurate O_2 and Li_2O_2 assays to separate discharge and charge stability limitations in nonaqueous Li−O_2 batteries. *J. Phys. Chem. Lett.* **4**, 2989-2993 (2013).
