# Supplementary material for: A versatile functionalized ionic liquid to boost the solution-mediated performances of lithium-oxygen batteries
Source: Nat Commun. 2019 Feb 5;10:602. doi: 10.1038/s41467-019-08422-8 (PMC6363722; doi:10.1038/s41467-019-08422-8)
Supplement: Supplementary file 3 — Description of Additional Supplementary Files [file 41467_2019_8422_MOESM3_ESM.pdf]

Description of Additional Supplementary Files

1. **Supplementary Movie 1 | The mechanism experiments demonstrating the interactions between reduced IL-TEMPO and oxygen.**
